# Epigenetic Viral Mimicry in Neuroinflammation: Retrotransposons, cGAS–STING and Microglial Hysteresis

**DOI:** 10.3390/medsci14060635

**Published:** 2026-10-02

**Authors:** Sorin Ioan Tudorache, Valentin Titus Grigorean, Adrian Vasile Dumitru, Maria Fulina, Matei Șerban, Răzvan-Adrian Covache-Busuioc, Corneliu Toader, Dan Dumitrescu

**Affiliations:** 1 Department of Preclinical Disciplines, Faculty of Medicine, Titu Maiorescu University, 040051 Bucharest, Romania; 2 Faculty of General Medicine, “Carol Davila” University of Medicine and Pharmacy, 050474 Bucharest, Romania; mateiserban@innbn.com (M.Ș.);; 3 Puls Med Association, 051885 Bucharest, Romania; 4 Department of Pathology, Faculty of Medicine, “Carol Davila” University of Medicine and Pharmacy, 030167 Bucharest, Romania; 5 Medical Doctoral School, Faculty of Medicine, Ovidius University of Constanta, 900470, Constanța, Romania; 6 County Clinical Emergency Hospital “Sf. Ap. Andrei”, 900591 Constanța, Romania; 7 Department of Neurosurgery, “Carol Davila” University of Medicine and Pharmacy, 050474 Bucharest, Romania; 8 Department of Vascular Neurosurgery, National Institute of Neurology and Neurovascular Diseases, 077160 Bucharest, Romania

**Keywords:** neuroinflammation, microglia, neurodegeneration, transposable elements, viral mimicry, cGAS–STING, LINE-1, epigenetics

## Abstract

Endogenous retroviruses (ERVs), LINE-1 elements, and other endogenously active transposable-element families are now being studied for their role as a potential interface between epigenetic control and innate immunity. Here, we discuss how locus-specific failures in repressive complexes containing ATRX–DAXX–H3.3 and TRIM28–SETDB1–H3K9me3 may allow these elements to enter into unique molecular states that include double-stranded RNA, DNA/RNA hybrid molecules, RNA–protein complexes, reverse-transcription products, and RNA. Each of these states differs from the others in terms of its persistence, structural characteristics, and accessibility to sensors such as MDA5, RIG-I, cGAS–STING, and ZBP1, as well as host restriction pathways. We also emphasize that simply expressing a retroelement does not imply that it is competent to bind to a sensor and/or undergo productive retrotransposition. Rather, a distinction needs to be made between intermediate stages in the process of retrotransposition and productive retrotransposition itself. Therefore, the evidence needed to assign a specific sensor to a particular retroelement product must be presented. Furthermore, persistent stress caused by retroelement activity could converge with multiple cellular processes, such as p53–CDKN1A/p21-mediated cell-cycle arrest, PML-mediated nuclear organization, and lysosomal or metabolism-based changes leading to long-lived microglial states; however, demonstrating path dependence after removing the initiating perturbation would be required to demonstrate true hysteresis. Finally, downstream activation of complement pathways could have effects on synaptic function via separate CR3-dependent, trafficking-based, and contact-regulatory mechanisms. Overall, we recommend a locus-specific, structure-specific, and time-course-resolved approach for determining how the various states of endogenously active retroelements cross the genome–immunity boundary and contribute to neuroinflammatory dysfunction.

## 1. Introduction: The Repetitive Genome as a Latent Neuroimmune Compartment

The first step is to understand what causes neuroinflammation and how endogenous retroelements (EREs), alongside inflammatory cytokines, complement components, nucleotides, and other mediators, contribute to the inflammatory process. In addition to understanding these contributing factors, we have to ask “How do ERE-derived molecular species acquire immunogenic significance?”. They cannot simply appear as danger signals; they must pass several biochemical barriers related to chromatin accessibility, transcription, processing, molecular structure, protein association, stability, reverse transcription, and subcellular localization [1,2]. Since subsets of EREs retain promoter activity, coding potential, repetitive structures, and the machinery needed to produce RNA, proteins, and nucleic-acid intermediates similar to those produced during viral replication, they are good candidates to serve as key substrate molecules at the genome–immunity interface [3].

Therefore, their impact on the neuroimmune system will depend on both their genomic location and expression level, as well as the physical state of the molecular species they generate and whether that state is accessible to cellular surveillance [4,5]. Due to the multitude of outcomes possible following loss of repression of a retroelement, it is critical to use precise terminology. Activation of a retroelement in this manuscript is used solely as a broad category and, when sufficient detail is available, is broken down into individual categories based on what has occurred [6]. Categories include derepression of chromatin, increased chromatin accessibility, autonomous transcription, RNA accumulation, translation, assembly of ribonucleoproteins (RNPs), ORF1p or ORF2p production, reverse transcription, cDNA generation, and successful genomic integration [7].

These categories are not equivalent: increased chromatin accessibility does not mean that transcription will occur; transcription does not imply translation; RNA accumulation does not indicate protein production; detection of ORF1p does not establish ORF2p expression or enzymatic competence; and reverse-transcribed DNA does not indicate completed retrotransposition [8,9]. Therefore, we define retroelement molecular-state conversion as the conversion of an ERE-derived product from one physically and biochemically distinguishable state to another. Molecular-state conversion does not imply that these molecular states must occur in a linear sequence. Additionally, the term supramolecular checkpoint herein refers to the requirement for coordinated assembly of multiple molecular components for conversion from one molecular state to another [10,11].

Lastly, we define microglial hysteresis as a conceptually testable description of path-dependent persistence wherein an altered state of microglia becomes partly uncoupled from the cause of that alteration (i.e., an insult) and continues to exist even though that cause has either diminished or been experimentally corrected. Persistence alone does not necessarily imply hysteresis [12].

The types of molecular products formed by various classes of EREs, and subsequently processed, can vary significantly. For instance, ERV-derived RNAs can form dsRNA that displays diverse immunological behaviors dependent on duplex length, mismatch density, RNA editing, secondary structure, protein occupancy, persistence, and intracellular localization. Conversely, LINE-1 RNA can assemble with ORF1p into RNPs and proceed toward genomic nicking and target-primed reverse transcription if ORF2p is present and enzymatically competent [13]. RNA–DNA hybrids represent yet another type of molecular species and should not be confused with canonical R-loop structures containing a displaced ssDNA strand [14]. The generation and resolution of these hybrid molecules depend on reverse-transcriptase activity, RNase H enzymes, topoisomerases, helicases, nucleases, transcriptional and replication dynamics, and DNA-repair pathways. Consequently, bulk measurements of retroelement RNA levels can still provide evidence of disruptions in genomic regulation, but they provide limited insight into the mechanism(s) responsible for the observed changes [15].

More informative parameters would include determination of which locus is activated; which physical type of molecule is formed; how long the molecule persists; in which compartment the molecule localizes; and whether the molecule converts into another type of molecular species, such as an RNP, RNA–DNA hybrid, or reverse-transcribed DNA [16].

Similarly high levels of precision are required when assigning specific innate sensors to retroelement-derived molecules. Cooperative assembly of MDA5 along sufficiently long and continuous dsRNA can support downstream signaling; conversely, terminal architecture and end accessibility influence RIG-I recognition [17]. Aberrantly exposed or reverse-transcribed DNA can become accessible to cGAS provided that it escapes degradation, chromatinization, or protective protein associations. However, structural compatibility with a receptor does not confirm direct biochemical association. Stronger evidence includes direct ligand–sensor interaction; recovery of receptor-associated nucleic acid; genetic epistasis; and separation-of-function experiments demonstrating selective effects on the proposed sensing pathway upon disruption of a defined molecular feature [18].

Therefore, relationships throughout this manuscript for which direct engagement has not been established are described using terms such as supported, consistent with, or proposed, rather than as definitive ligand–sensor pairs.

Containment can also occur prior to canonical sensing. An instructive example is STING. While STING has an established role downstream of cGAS signaling, it can also interact with LINE-1 ORF1p, facilitating trafficking through ER-associated and endolysosomal compartments and thereby reducing persistence of LINE-1 machinery before significant amounts of reverse-transcribed DNA accumulate [19]. We refer to this layer as pre-sensor restriction, emphasizing that innate pathways play roles both in maintaining the genome–immunity boundary and in responding to breaches of that boundary [20].

This dual positioning also emphasizes that activation of an innate pathway should not automatically be interpreted as evidence for a single downstream sensing mechanism. There is a fundamental distinction between acute viral mimicry and chronic pathology [21]. Nucleic-acid sensing can induce interferon-dependent programs involving RNA editors, nucleases, helicases, restriction factors, and trafficking pathways, thereby increasing containment capacity [22]. Chronic dysfunction becomes more likely when the generation and persistence of abnormal retroelement-derived species exceed the collective capacity for editing, degradation, sequestration, trafficking, repair, and chromatin restoration [23]. Stress caused by retroelements may converge with genomic nicking, unresolved RNA–DNA hybrids, transcription–replication conflicts, and DNA-damage signaling [24]. While these events represent partially overlapping processes whose contributions may vary depending on cell state, molecular intermediate, and disease context, they should not be assumed to constitute a single obligatory cascade [25].

Within this framework, microglial hysteresis is considered a testable working hypothesis rather than an established mechanism. Persistent p53–CDKN1A/p21–RB signaling, PML nuclear-body organization, lysosomal remodeling, and metabolic adaptation have been observed in prolonged altered cellular states in previous studies and are compatible with progressive loss of reversibility. However, these observations individually do not demonstrate hysteretic behavior [26].

To truly demonstrate path dependence would require correction or removal of the initial perturbation following development of an altered microglial state; verification that the upstream molecular abnormality has returned toward baseline; and persistence of downstream state characteristics following correction or removal of the initial perturbation [27]. Section 5 addresses candidate molecular mechanisms supporting sustained changes in microglial states, whereas Section 7 describes temporal reversal experiments capable of differentiating delayed recovery from true molecular memory.

Disruption of normal regulation within repetitive genomic regions can also propagate beyond the cell in which the initial disruption occurs. Our previous study indicated that loss of SETDB1-mediated repression in neurons was accompanied by ERV-derived RNA–DNA hybrids, increased C4b, and downstream microglial and synaptic abnormalities [28]. These data suggest an epigenome-to-complement link in this experimental context. However, the biochemical relationship between specific retroelement-derived hybrid species and complement activation remains incompletely understood. Complement can operate through distinct mechanisms. C3-derived opsonins, particularly iC3b, can engage microglial CR3, facilitating capture of complement-tagged synaptic material, whereas C4-associated pathology can intersect with neuronal SNX27–Rab11-dependent AMPAR trafficking and CR3-dependent regulation of microglial contact behavior [29].

Therefore, “complement-mediated synapse elimination” lacks clear mechanistic definition unless the relevant complement species, receptor or trafficking pathway, and cellular outcome are defined, as well as whether dysfunction results from engulfment, receptor missorting, altered microglia–synapse interactions, or a combination thereof [30].

These data collectively support consideration of the repetitive genome as a latent neuroimmune compartment bounded by partially overlapping layers of control. Chromatin architecture dictates locus accessibility; pathways for RNA processing and nucleic-acid resolution dictate molecular structure; proteins encoded by retroelements regulate progression toward RNP assembly and reverse transcription; pre-sensor restriction dictates ligand availability; innate sensors discriminate among visible molecular species; and checkpoint, glial, complement, and synaptic mechanisms dictate whether the response resolves or persists [31,32]. Therefore, viral mimicry is not a singular pathway but an emergent condition in which endogenous genomic information develops physical properties compatible with pathogen-oriented surveillance [33].

Two interpretative principles guide this manuscript. Mechanistic conservation and locus conservation are recognized as fundamentally distinct. Processes including heterochromatin maintenance, RNA editing, nucleic-acid sensing, DNA-damage signaling, and complement biology may be broadly conserved across mammals; however, active ERV families, intact LINE-1 copies, promoter architectures, insertion polymorphisms, and locus-specific regulatory environments are highly variable among species. Studies utilizing rodents are therefore used primarily to identify potentially conserved molecular mechanisms rather than to assume equivalent retroelement repertoires between rodents and humans [34].

Additionally, strong caution is exercised when assigning ligand–sensor relationships to specific retroelement-derived molecules; determining whether persistence of a microglial state represents true path dependence; extrapolating findings between species; and establishing causal ordering between cells [35].

The narrative literature review carried out for this paper was based on a targeted literature search, last updated on 22 September 2026, for which the main source was PubMed/MEDLINE. Searches were also supplemented by backward and forward citation screening of primary research studies and contemporary reviews concerning the subject matter of the searches. The search terms used included endogenous retroviruses, LINE-1, retrotransposons, ATRX–DAXX, TRIM28–SETDB1, H3K9me3, double-stranded DNA, dsRNA, RNA–DNA hybrids, reverse transcription, MDA5, RIG-I, cGAS–STING, ZBP1, microglial remodeling, cellular senescence, PML, complement activation, CR3, synaptic transport, and synaptic pathology. Search terms were employed individually or in combination as necessary to identify relevant mechanisms. When identifying supporting studies for claims regarding mechanism, primary studies considered to be mechanistic, including genetic, biochemical, structural, and disease-relevant studies, were prioritized. Of primary interest were studies that identified molecular identity, showed direct physical interaction(s), demonstrated pharmacological or genetic dependence, established temporal relationship(s) between event(s), provided rescue or separation-of-function data, and/or demonstrated functional effects. Variability in results reported by studies was assessed relative to variables such as the species studied, cell type and developmental stage, experimental system or methodology employed to assess effect(s), treatment regimen(s) utilized to generate effect(s), and methodology employed to measure the effect(s) produced. Results derived from biochemical systems, cell culture, ex vivo preparations, animal models, human cells or tissues, and human observational studies are described in light of the methodological characteristics of each type of study. Results derived from reductionist systems or non-human models are not considered, by themselves, evidence of human disease causality. As this is a narrative literature review focused on mechanistic evidence rather than a systematic review or meta-analysis, the literature search strategy was utilized to select representative and mechanistically relevant evidence rather than to provide a quantitative summary of all available literature.

Therefore, this manuscript explores the genome–immunity boundary from locus-selective ATRX–DAXX–H3.3/TRIM28–SETDB1–H3K9me3 repression through formation of distinct RNA, RNP, RNA–DNA hybrid, and reverse-transcribed states; ligand accessibility; innate-sensor discrimination; pre-sensor restriction; persistent microglial remodeling; and complement-associated synaptic dysfunction. The primary questions are therefore not simply whether a retroelement is activated, but rather which locus escapes; which molecular state is achieved; which host checkpoint fails to restrict it; which surveillance pathway recognizes it; and which transition(s) are experimentally necessary for neural dysfunction.

## 2. Locus-Selective Architecture of the Epigenetic Firewall

The architecture of the epigenetic firewall is determined by the way the various locus-specific interrelationships between chromatin-modifying proteins interact with each other. As individual proteins (e.g., ATRX, DAXX, TRIM28, SETDB1), they are involved in a variety of processes leading to repression of particular types of repetitive sequences. However, as a collective unit, they comprise a common repressive architecture necessary to maintain the repressed status of several repetitive sequences [36]. The greater the number of regulatory molecules controlling a given nucleosomal substrate, the greater will be the degree to which that substrate’s chromatin state is regulated. It is anticipated that if the repressive architecture provided by ATRX, DAXX, TRIM28, and SETDB1 were to be disrupted, a disproportionately greater regulatory burden would likely fall on those genetic elements sufficiently autonomous to generate RNA and/or protein and undergo reverse transcription [37].

### 2.1. ATRX–DAXX–H3.3 Couples Chromatin Recognition to Repressive Histone Renewal

The ADD domain of ATRX recognizes a repressed chromatin state by detecting both unmethylated H3K4 and trimethylated H3K9. By utilizing combinations of histone modifications, ATRX can recognize a repressed nucleosome without relying on a single histone modification [38].

Recent studies have expanded upon the ADD domain’s ability to detect repressed nucleosomes. ATRX binds to the positively charged R40 residue located in the L12 loop of macroH2A via an acidic surface near the H3K9me3-recognition region of the ADD domain [38]. Additionally, ATRX participates in a cation–π interaction between the positively charged guanidinium group of macroH2A R40 and the aromatic amino acid residue Y203 of ATRX. Together, residues Y203, H189, and E252 create a surface able to bind either H3 or macroH2A. Therefore, H3 and macroH2A may both contact overlapping areas of the ADD domain of ATRX. Models constructed from three-dimensional structures of CHD4 demonstrate molecular mimicry between CHD4 and H3-like geometry; therefore, K261 is located analogously to H3K4, and R271 is positioned similarly to the H3K9me3-recognition region [39]. Due to its ability to recognize multiple ligands, ATRX can take advantage of the chemical characteristics of histone tails, histone variants, and physical interactions with non-histone chromatin-remodeling complexes to recognize discrete regions. Local molecular composition of chromatin could contribute to where ATRX is directed toward repetitive DNA rather than localization being determined solely by H3K9me3 density. Therefore, chromatin containing H3K9me3-modified histone tails, macroH2A, and NuRD/CHD4 complexes could potentially compete or cooperate in directing ATRX localization, regulating chromatin-remodeling kinetics, and recruiting additional partners [40].

Following recognition by ATRX, DAXX-mediated histone replacement results in histone-state renewal. The histone-binding domain of DAXX specifically binds H3.3–H4 dimers. Biochemical analyses of DAXX-deficient microglia indicate a clear dissociation: H3K9me3 levels are reduced at certain LTRs; however, H3.3 occupancy remains relatively stable at these sites [41]. The absence of H3.3-containing nucleosomes itself cannot account for these biochemical alterations; rather, DAXX determines the biochemical conditions under which H3.3-containing nucleosomes are incorporated or maintained. These conclusions are supported by differences among histone-variant chaperoning pathways: removal of HIRA does not result in biochemical alterations equivalent to those resulting from removal of DAXX; removal of H3.3 results in only partially equivalent biochemical alterations compared with removal of DAXX. Thus, the same histone variant can perform different biological functions depending upon the chaperone pathway utilized for its introduction [42].

Furthermore, DAXX forms a direct physical association with SETDB1 and members of the SUV39 family of H3K9 methyltransferases, providing a mechanistic connection between delivery of H3.3–H4 dimers and establishment or re-establishment of repressive methylation before or during deposition. Nucleosome turnover at loci undergoing replication-independent nucleosome exchange therefore does not necessarily result in loss of heterochromatic identity, provided that replacement histones are introduced together with enzymes capable of establishing or restoring H3K9me3 [43]. ATRX recognizes the repressively modified nucleosomal environment; DAXX supplies H3.3–H4 dimers; and associated methyltransferases establish or restore the chemical state of newly exchanged nucleosomes. Therefore, repressively modified nucleosomes can be continuously maintained across multiple cycles of histone replacement and establishment or restoration of repressive nucleosomal states [44].

This repressive nucleosome-replacement process also interfaces with G-quadruplex (G4) DNA, thereby increasing the range of the epigenetic firewall to include structurally challenging genomic regions. G4 structures occur preferentially in G-rich sequence contexts and can act as barriers to DNA replication or transcription unless properly managed [45]. ATRX physically interacts with G4-containing regions, and loss of ATRX results in elevated G4 accumulation at newly synthesized DNA. Importantly, ATRX activity and DAXX-mediated deposition of H3.3 contribute to protection against G4-associated replication stress; conversely, loss of ATRX or DAXX reduces H3K9me3 at ATRX-bound G4 regions [46]. Collectively, these findings suggest that ATRX–DAXX–H3.3 contributes to maintaining structurally challenging DNA within a closed chromatin environment rich in H3K9me3 rather than acting simply as a general histone-replacement mechanism. Additional evidence supporting this association comes from studies performed with actively dividing cell populations; whether similar protective associations exist in post-mitotic neuronal populations through transcription-coupled chromatin stabilization and resolution of structural stresses generated by ongoing transcription remains unclear [47].

Therefore, this distinction is critical when comparing proliferative neural stem/progenitor or glial populations with post-mitotic neurons. Neural stem/progenitor and proliferative glial populations are required to maintain heterochromatin composed of repetitive elements during DNA replication, replication-fork progression, and nascent-chromatin assembly. Post-mitotic neurons utilize replication-independent histone turnover to maintain their chromatin organization over extended periods despite continuing transcriptional activity [48]. Disruption of ATRX–DAXX-dependent surveillance mechanisms would therefore be expected to generate replication-associated structural stress in proliferative cells and transcription-associated chromatin instability in post-mitotic neurons. Experimental evaluation is required to determine whether such disruption causes comparable instability in glial cells, neural progenitor cells, and differentiated neurons [36].

Lastly, histone replacement shows significant selectivity for repeat-family loci. IAP-related loci generally exhibit high basal levels of H3K9me3 compared with most other LTR families and display high sensitivity to DAXX deficiency. Conversely, ERV1, ERVK, and other LTR families exhibit variable dependence on ATRX–DAXX-based maintenance. Variables contributing to this dependence include promoter architecture; CpG content; age of repeat families; specificity of KRAB-ZFP targeting; proximity of enhancer elements; accessibility to transcription factors; frequency of nucleosome-exchange events at each locus; and overall organization of local chromatin [49]. Table 1 summarizes the fundamental principles by which ATRX–DAXX couples recognition of the nucleosomal environment to H3.3 turnover and the establishment or re-establishment of H3K9me3, thereby maintaining repeat-family-selective heterochromatin identity.

### 2.2. SETDB1–H3K9me3 Propagates Repression into HP1, H4K20me3 and CpG Methylation

Whereas SETDB1 effectively establishes repressive states marked by H3K9me3, H3K9me3 itself does not constitute the final component of this system. Instead, after establishment, H3K9me3 provides a site for HP1 binding and indirectly supports recruitment of SUV420H2 to establish H4K20me3 and maintain DNA methylation. These repressive layers can reinforce one another. Together, the combination of these repressive layers results in a multilayered repressive state [61].

Examples of the relationship between H3K9me3 and repression in LINE-1 elements include younger LINE-1 families that have undergone less degeneration and exhibit relatively consistent H3K9me3 enrichment across their structures. Older families that have undergone greater degeneration show less consistent H3K9me3 enrichment. Because repression focuses primarily on LINE-1 copies most likely to retain a functional 5′ promoter region, intact open reading frames, and retrotransposition competence, only a few closely related full-length loci retaining these properties are selectively reactivated following loss of SETDB1 [62]. A second key neural context in which this occurs is represented by human neural stem/progenitor cells. Following SETDB1 deletion, loss of H3K9me3 occurs in conjunction with selective derepression of young LINE-1 elements and reduction in CpG methylation at the promoters of these elements [63]. While locus sensitivity appears to depend upon the genomic location of LINE-1 integration, no single linear KRAB-ZFP–TRIM28–SETDB1 recruitment mechanism fully explains SETDB1 recruitment. Smaller effects are observed following deletion of either TRIM28 or MORC2 compared with deletion of SETDB1 at young LINE-1 loci. It therefore appears that multiple upstream pathways may recruit SETDB1 or support the enzymatic activity of SETDB1. Therefore, SETDB1 represents a point of enzymatic convergence within a broader chromatin architecture rather than simply a terminal enzyme in a single recruitment pathway [64].

Following deletion of SETDB1, responsive LINE-1 promoters undergo significant chromatin remodeling. The amount of H3K9me3 present at responsive LINE-1 promoters decreases; conversely, the amount of H3K4me3 increases; CpG methylation decreases; and 5-hydroxymethylcytosine (5hmC) increases [65]. These findings collectively support the following mechanistic model:

Decrease in H3K9me3 → increase in 5mC oxidation/5hmC → decrease in CpG methylation → increase in H3K4me3 → promoter activation

This sequence should not be interpreted as an obligatory sequence of events, but rather as a mechanistically supported model illustrating how H3K9me3 may help protect CpG methylation from erosion rather than simply colocalizing with CpG methylation. When H3K9me3 is sufficiently diminished, methylated CpG sites may become increasingly susceptible to oxidation and demethylation. This may allow cooperative progression toward transcriptional competence [66].

Results from experiments using human neural progenitors indicate that young LINE-1 promoters lose CpG methylation and gain 5hmC following SETDB1 depletion [67].

In addition, HP1 proteins extend the repressive environment by interacting with H3K9me3 through their chromodomains and converting what would otherwise be a covalently modified histone into a multivalent protein complex. Upon interaction with H3K9me3, HP1γ utilizes its chromoshadow domain to interact with SUV420H2 [68]. Interaction with HP1γ enables deposition of H4K20me3 through hydrophobic surface contacts involving HP1γ residues I165, F167, and L172. Therefore, heterochromatin formation may depend on specific residue-level protein–protein interactions rather than generalized chromatin compaction alone. HP1β is also involved in maintaining DNA methylation [69].

Therefore, the interdependencies among these architectural components can be represented schematically as follows:

SETDB1 → H3K9me3 → HP1 association → SUV420H2 recruitment → deposition of H4K20me3

Furthermore, HP1 facilitates maintenance of CpG methylation as well as supporting other types of repressive marks and higher-order chromatin organization [70].

As previously mentioned, the relationships described above create local epigenetic cooperation. The presence of H3K9me3 at a particular genomic location creates opportunities for HP1 association at that location. Association of HP1 with a particular location can facilitate higher-order chromatin organization as well as promotion of other types of repressive marks [71]. Restriction of transcription-factor access to a particular location by DNA methylation creates an environment favorable to retention of repressive complexes. Conversely, if H3K9me3 falls below a locus-specific threshold, HP1 association may decline; H4K20me3 and DNA methylation stability may decrease; TET-mediated oxidation may increase; active histone marks may accumulate; and transcription factors may gain access to the locus. Thus, instead of losing repression proportionally following loss of one modification, a locus can transition nonlinearly between distinct chromatin states [72,73].

Nonetheless, determining whether inflammatory responses converge with this architecture requires consideration of potential causal relationships. For example, maternal immune activation has been associated with altered H3K9me3 organization and transposable-element expression, although recently published data suggest overlapping consequences of maternal immune activation and neuronal SETDB1 deficiency involving ERV-derived RNA–DNA hybrid formation in neurons, neuronal C4b induction, and subsequent microglial synaptic remodeling [74,75]. Although these studies represent disease-relevant examples in which inflammatory stress and disrupted retroelement regulation converge on shared downstream states, they do not conclusively establish whether inflammatory exposure directly impairs SETDB1 enzymatic activity. Therefore, the most conservative conclusion is that inflammatory stress can perturb the broader H3K9me3–retroelement-containment landscape and that contributions provided by SETDB1 depend on cell type, developmental stage, and inflammatory context [76].

Similarly, several ERV loci exemplify analogous mechanisms through differing regulatory architectures. Deletion of SETDB1 can lead to both decreased levels of H3K9me3 and increased levels of H3K27ac at some ERV loci. As such, an initially repressed retroviral element can develop enhancer-like properties. Additionally, altered expression of genes proximal to an ERV element that has undergone reactivation following SETDB1 deletion does not necessarily correlate with concurrent loss of H3K9me3 at their respective promoters. Therefore, regulatory dysfunction caused by SETDB1 deletion can be contained within the ERV loci themselves rather than occurring nonspecifically through spreading of euchromatic states [77].

### 2.3. Locus-Selective Escape Converts Retroelements into Transcriptional and Regulatory Units

The process that determines whether a retroelement will move from a repressively bound state to a more unstable boundary state from which it may escape repression involves both intrinsic properties of the retroelement and extrinsic properties of its local environment [10]. These properties include the evolutionary age of the retroelement and the degree of its sequence conservation; its orientation within the local genomic context; CpG density; promoter competence; rate of nucleosome turnover; long-range spatial genome structure; replication timing; association with the nuclear lamina; proximity to enhancers; and the presence of transcription-factor motifs. Each of these intrinsic and extrinsic features affects the ease of escaping locus-specific barriers and gaining access to the transcriptional apparatus [78].

Escape from locus-specific barriers in DAXX-deficient microglia correlates with changes in cellular regulatory identity in newly accessible regions. Newly accessible genomic regions are significantly enriched for AP-1 motifs, whereas newly accessible intergenic LTRs are enriched for motifs associated with PU.1 [79]. Although motifs from these families represent two fundamentally different types of regulatory information, they cannot be used interchangeably. For example, the AP-1 family links chromatin accessibility to stress-responsive signal-transduction pathways, whereas PU.1 contributes heavily to enhancer landscapes that define microglial lineage identity. Therefore, when a previously occluded repetitive genomic region becomes accessible, a dormant LTR can acquire the ability to serve as a substrate for transcription-factor-mediated activity [80].

However, motif enrichment does not indicate physical occupancy of a site by a transcription factor without direct evidence of protein–DNA interaction.

Accessibility of newly available LTRs occurs in a non-random manner. They frequently appear adjacent to immunoregulatory and interferon-responsive genes. Typically, accessibility is reduced adjacent to adult homeostatic regulatory regions including Sall1, P2ry12, Cx3cr1, and Mafb, including the Sall1 super-enhancer. Instead of widespread chromatin decondensation, these data suggest that regulatory potential is redistributed away from adult homeostatic programs toward developmental and/or inflammatory/reactive programs [81].

Because these data were obtained using well-defined experimental systems, redistribution of regulatory potential analogous to that observed in this study should not be assumed in other microglial states, neurological disease models, or species without direct verification [82].

When an element loses portions or all of its repressive architectural structure, it can take on two functionally distinct roles. It can produce RNA or subsequent nucleic-acid intermediate(s) that have effects outside its original location via a trans-acting mechanism. Alternatively, it can act as a promoter or enhancer at or near its original genomic location via a cis-regulatory mechanism. Even though these two mechanisms produce distinct outcomes, they can also reinforce one another [83]. Retroelement-derived ligands can enhance signaling pathways mediated by AP-1-, IRF-, and STAT-associated proteins; furthermore, newly accessible repetitive sequences can create additional sites where transcription factors involved in these pathways can potentially bind. Recruitment of cofactors and histone acetyltransferases can then increase H3K27ac levels and thereby stabilize the newly accessible region. Therefore, loss of repression may lead to self-reinforcing chromatin transitions; however, each event must be demonstrated individually for each locus rather than assumed universally [84,85].

There are several properties of a given LTR that determine whether it can function as a promoter or enhancer. One of the most important is its evolutionary history as an effective recruiter of host transcriptional machinery. Many retroviral regulatory sequences contain high densities of host transcription-factor motifs. Therefore, genomes limit both viral-like transcription and latent regulatory information encoded within these repeats through heterochromatic organization [86].

Similarly, young LINE-1 elements exhibit related principles because their 5′ UTRs retain autonomous promoter activity. Furthermore, some LINE-1 elements preserve intact open reading frames encoding ORF1p and ORF2p. Depletion of SETDB1 in cultured cells demonstrates that certain subsets of L1HS/L1PA loci are activated while most comparable copies remain inactive; concomitant loss of H3K9me3 and CpG methylation together with acquisition of H3K4me3 has also been shown [87].

Similarly, depletion of SETDB1 in human neural stem/progenitor cells preferentially activates evolutionarily young LINE-1 elements rather than globally activating the entire LINE-1 repertoire [88]. Importantly, family-wide expression can obscure important biological differences among individual loci; transcription from numerous defective copies is not mechanistically equivalent to transcription from fewer competent copies capable of producing ORF1p, ORF2p, and reverse-transcription products [89].

Therefore, three general categories of locus escape can be identified. Individual repeats can lose some portion of their repressive architecture yet remain transcriptionally inactive; alternatively, they can become transcriptionally active without a corresponding change in regulation of genes adjacent to them; or they can undergo regulatory capture, whereby they lose repressive marks, acquire activating chromatin attributes, become occupied by transcription factors, and contribute to regulation of adjacent genes. These states do not necessarily occur in sequential order, nor do they require equal disruption of SETDB1 activity, HP1 residency, DNA methylation, or availability of transcription factors [90].

The threshold model provides a reasonable synthesis of prior observations. Repetitive loci are regulated by the cumulative effects of ATRX recognition; DAXX-mediated histone exchange; SETDB1 catalytic activity; H3K9me3 density; HP1 residency; H4K20me3; CpG methylation; and nuclear compartmentalization, counteracted by processes including histone turnover, DNA demethylation, structural stress, and transcription-factor occupancy. A locus may exist well within a repressed state or near a metastable boundary. Disruption of one layer of regulation can destabilize multiple interacting layers, making a locus more susceptible to transitioning toward transcriptional competence [91].

This threshold model represents a mechanistic integration rather than an inherent characteristic of all repetitive loci; an essential prediction of the model is that comparable upstream disruptions should generate disparate responses among individual repeat copies [92].

Ultimately, the output produced by this architecture is not generalized heterochromatin loss but rather the selective conversion of specific repetitive loci into either transcriptional or regulatory units. After a locus crosses this boundary, its biological consequences depend greatly upon the molecular species produced by that locus: single-stranded RNA (ssRNA); double-stranded RNA (dsRNA); a LINE-1 RNP; an RNA–DNA hybrid; or reverse-transcribed DNA [93]. Consequently, Section 3 explores the biochemical transformations among the distinct molecular states created by escaped loci.

## 3. Retroelement Molecular-State Conversion

Transcription of retroelements will generate intermediate forms that are biologically functional based upon their structural characteristics, associations with proteins, ability to persist in their original location or elsewhere, enzymatic processing, as well as quantity. These intermediate states can undergo degradation, form dsRNA structures, develop an RNP particle, combine to create hybrids that include RNA and DNA, or undergo reverse transcription, etc.; therefore, expression at the locus level is distinguishable from the development of a structurally and spatially competent downstream molecular state [5,94].

### 3.1. ERV-Derived dsRNA: From Transcriptional Escape to Structurally Competent RNA

When complementary strands of RNA derived from ERVs persist as stable duplexes for prolonged periods, their structural characteristics differ substantially from those associated with single-stranded RNA. Bidirectional transcription generates complementary strands of RNA, whereas unidirectional transcription generally produces only one strand. Stable duplexes can arise through bidirectional transcription and/or from overlapping transcription units, inverted-repeat structures, and intramolecular folding [60]. Most duplexes are heterogeneous; that is, they do not display equal amounts of continuous helix and frequently include internal bulges, mismatched base pairs, and non-standard terminal configurations. The distinction between whether duplexes serve as substrates undergoing routine processing or possess sufficient structural coherence to act as scaffolds for higher-order sensor complexes ultimately depends on both their underlying structure and the nature of modifications made to the duplexes after transcription [95].

Modification of adenosine residues to inosine through ADAR1 editing alters local pairing interactions, stacking interactions, and helical regularity, thus affecting this transition without eliminating the RNA. Structural integrity, including continuity of A-form duplex RNA, can therefore be reduced. Because the predominantly nuclear ADAR1 p110 isoform can edit newly synthesized repetitive dsRNA, whereas the interferon-inducible ADAR1 p150 isoform has substantial cytoplasmic activity and can edit exported repetitive dsRNA during an immune response, whether editing occurs does not by itself determine immunological competence. Whether sufficient residual segments of incompletely edited duplex RNA remain intact and structurally organized to support assembly of higher-order sensors is also determinative [96].

Further limitations on RNA longevity limit access to subsequent downstream molecular states. Although decapping mediated by DCP2, 5′→3′ exonucleolytic activity mediated by XRN1, degradation mediated by the RNA exosome, RNA helicases, and RNP sequestration limit the time available for continued maturation of a transcript, stabilization of RNA and biological competence are not equivalent. Modification of pathways responsible for turnover and remodeling of RNPs can result in increased levels of either LINE-1 RNA or encoded protein without corresponding increases in productive LINE-1 retrotransposition, demonstrating that the kinetics of RNP assembly are controlled by mechanisms maintaining RNA quality rather than merely by transcript quantity [97]. Therefore, only that portion of retroelement RNA attaining sufficient structural organization to participate in downstream biological activities for sufficient durations defines the biologically relevant population. While abundant levels of a transcript do not guarantee functional competence, for example when predominantly fragmented, heavily edited, or sequestered populations prevail, even low-abundance transcripts can make a dominant contribution to signaling events if sufficient numbers of long-lived duplexes are present. Therefore, before considering retroelement-derived RNAs as candidate initiating ligands, demonstration of their structural organization is required. Structural competence alone, however, does not establish direct engagement of a particular innate sensor [98].

### 3.2. LINE-1 RNP Assembly and ORF2p-Mediated Target-Primed Reverse Transcription

The conversion of LINE-1 RNA to an autonomously functioning ribonucleoprotein complex (RNP) is a more complicated biochemical process. This occurs primarily because LINE-1 RNA is translated to generate ORF1p and ORF2p. ORF1p possesses four main regions: an N-terminal region, a coiled-coil oligomerization region, an RNA-recognition motif (RRM), and a C-terminal nucleic-acid-binding region. By forming trimers via its coiled-coil oligomerization region, ORF1p generates a multivalent scaffold capable of acting as both a nucleic-acid chaperone and nucleic-acid-binding protein. The propensity of ORF1p to associate predominantly with the LINE-1 RNA from which it was translated also contributes to the strong cis preference of LINE-1 [99].

Therefore, LINE-1 represents another example of a supramolecular checkpoint. Successful execution of LINE-1 activity depends upon the presence of RNA bound by ORF1p, sufficient quantities of ORF1p relative to its RNA, the presence of ORF2p, appropriate compartmentalization within the cell, and maturation of the RNP complex. Because ORF1p levels can increase independently of productive retrotransposition, productive retrotransposition cannot be inferred solely from increased ORF1p abundance [24].

ORF2p contains the enzymatic activities required for LINE-1 retrotransposition. Its N-terminal apurinic/apyrimidinic endonuclease-like domain cleaves one strand of genomic DNA. Its reverse-transcriptase domain uses LINE-1 RNA as a template to synthesize cDNA. The two enzymatic activities are connected through intervening and C-terminal domains that form numerous interactions with the enzymatic core [100]. Although mutations disrupting certain of these interdomain interactions can inhibit first-strand synthesis or subsequent retrotransposition events, they do not necessarily block all activity exhibited by ORF2p. Therefore, ORF2p acts as an integrated molecular machine whose constituent parts functionally interact with each other rather than acting independently [101].

When ORF2p cleaves a T-rich genomic target sequence, creating a free 3′-OH group, target-primed reverse transcription begins. Afterward, base pairing occurs between this exposed target DNA and the poly(A) tail of LINE-1 RNA to create the primer–template junction from which cDNA synthesis begins. Thus, chromosomal DNA itself serves as the primer for reverse transcription [102].

Structural models of this reaction indicate that ORF2p can bind target DNA, LINE-1 RNA, and nascent cDNA simultaneously. During cDNA synthesis, the reverse-transcriptase core accommodates the growing RNA–DNA hybrid, while neighboring domains position the upstream DNA. Catalysis is favored when substrates possess a single-stranded 5′ DNA segment contiguous with double-stranded DNA, resembling transient DNA configurations that can occur during replication or repair. In addition to primary-sequence considerations, target-site selection may therefore also depend on DNA topology [102].

Completion of retrotransposition is much less efficient than initiation. Completion involves second-strand cleavage, removal or degradation of RNA, second-strand synthesis, and repair of junctions. These latter processes depend on incompletely understood host machinery, and reverse transcription frequently terminates prematurely. Frequent 5′ truncation of inserts reflects this inefficient completion. Additionally, ORF2p activity may terminate with genomic nicks, incompletely synthesized cDNA, unresolved RNA–DNA hybrids, or aberrantly repaired products before productive retrotransposition is completed [103].

**Figure 1:**
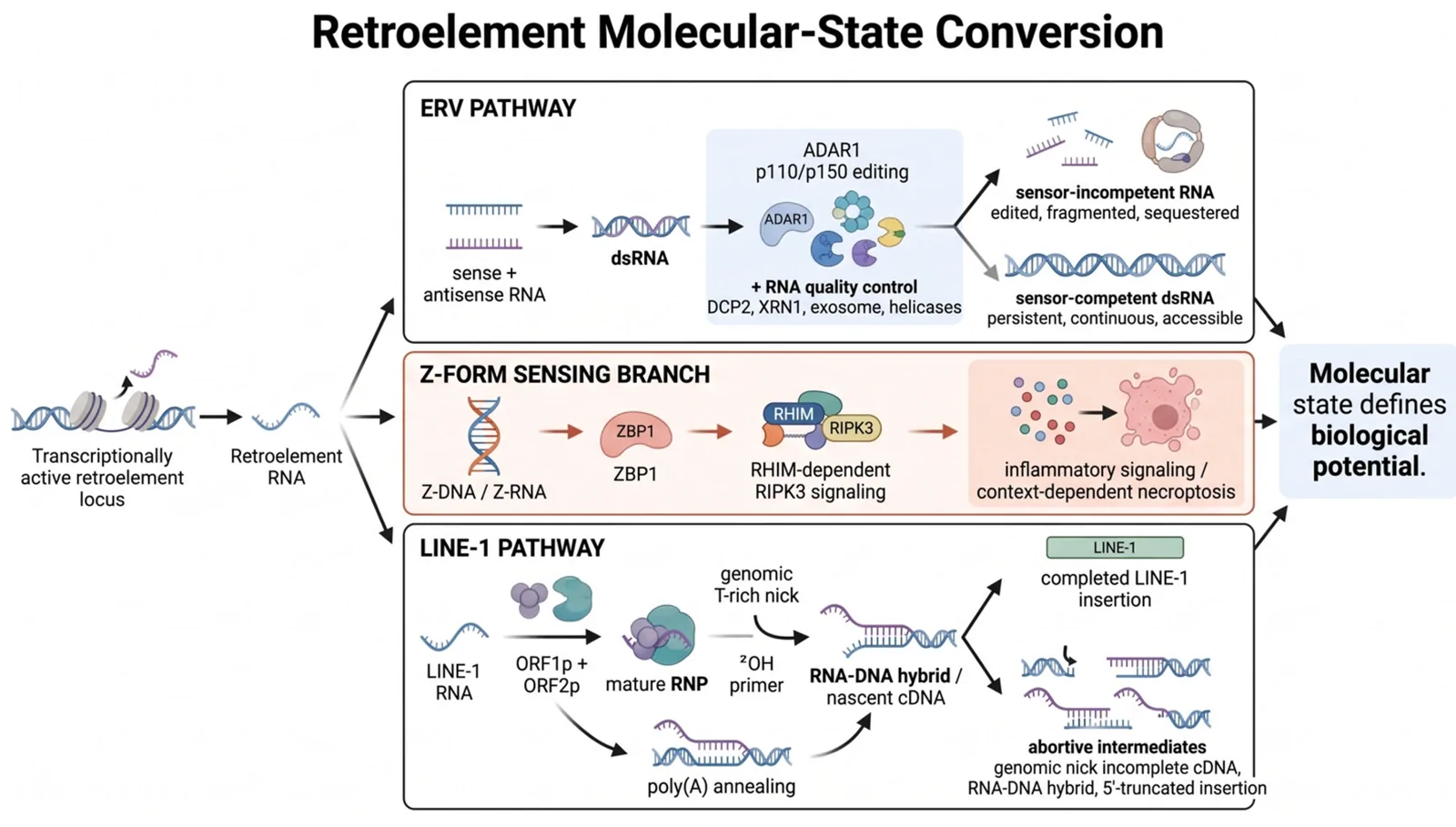
Retroelement molecular-state conversion. Transcriptionally active retroelements generate molecular states with distinct biological competence. ERV-derived transcripts can form dsRNA whose persistence is shaped by ADAR1 editing and RNA-quality-control pathways, whereas LINE-1 RNA assembles with ORF1p/ORF2p and can undergo target-primed reverse transcription. In parallel, retroelement-derived nucleic acids can adopt left-handed Z-RNA or Z-DNA conformations capable of engaging ZBP1 and context-dependent RHIM-associated inflammatory signaling. Productive insertion represents only one endpoint; abortive reactions generate RNA–DNA hybrids, incomplete cDNA, and genomic lesions that may themselves contribute to neuroimmune stress.

These final states constitute mechanistically distinct termination points. ORF1p functions as a nucleic-acid chaperone that supports assembly and stabilization of RNP complexes; ORF2p executes endonuclease and reverse-transcriptase reactions; cDNA indicates that reverse transcription has proceeded; and only newly generated genomic insertions demonstrate completed productive retrotransposition. Lastly, molecular stress relevant to neural pathology may arise at several stages of LINE-1 activity, including persistence of RNP complexes, generation of genomic nicks, production of RNA–DNA hybrids, or premature termination of DNA synthesis. As such, estimates of productive integration frequency may substantially underestimate the total molecular stress resulting from LINE-1 activity [104]. A schematic overview is shown in Figure 1, highlighting how retroelement transcription can diverge into structurally distinct ERV- and LINE-1-derived intermediates with different capacities for persistence, sensing and genome stress.

### 3.3. RNA–DNA Hybrids and Abortive cDNA as Transitional Neuroimmune Substrates

One of the primary changes in molecular state that can occur during retroelement activity is formation of RNA–DNA hybrids. Hybrids represent an important component of RNA biology because they bridge RNA processing and utilization pathways, reverse transcription, and genome maintenance. To differentiate a standard RNA–DNA duplex from a “canonical” R-loop, in which the hybridized region of RNA–DNA is accompanied by a displaced complementary DNA strand, it is important to recognize that the displaced DNA strand can function as an additional substrate for binding proteins including, but not limited to, RPA, nucleases, helicases, and enzymes involved in DNA-damage repair [105].

Hybrids can be created in at least two ways during retroelement activity. Nascent cDNA formed as a result of reverse transcription initiated by LINE-1 elements remains paired with its RNA template during synthesis. Thus, this necessarily creates an RNA–DNA hybrid intermediate. Additionally, transcripts derived from ERVs can form hybrids with complementary DNA sequences through co-transcriptional rehybridization and, in contexts in which reverse transcription occurs, through reverse-transcription-dependent mechanisms. Moreover, hybrids can be formed through more than one biochemical process. Therefore, identification of hybrids alone cannot provide sufficient information regarding the biochemical mechanism through which they were generated [106].

In addition, detection of hybrids using S9.6-based methods does not independently establish the presence of a canonical R-loop. Rather, additional orthogonal validation, particularly RNase H sensitivity together with appropriate structural controls, is necessary when discrimination between the mechanistic origins of hybrid structures is important [107]. Regardless of structural characteristics, many hybrids are relatively short-lived. Extended RNA–DNA duplexes can be removed through the actions of RNase H1. While RNase H2 plays a role in hybrid metabolism as well as removal of ribonucleotides incorporated into genomic DNA, its functions differ from those of RNase H1. Similarly, whether RNA reanneals to complementary DNA sequences is influenced by topoisomerases I and II, which regulate torsional stress within DNA [108].

Furthermore, resolution of intermediate structures may occur through their dismantling or conversion into DNA lesions through various helicases, nucleases, and enzymes involved in DNA-damage repair. Therefore, the potential pathological consequences associated with a particular hybrid will depend upon: (1) how rapidly the hybrid is formed; (2) how long it persists; (3) its relationship to other genomic features; and (4) how efficiently it is resolved [109].

Disruption of local DNA topology caused by longer-lived hybrids may impede either transcription or replication. The presence of exposed single-stranded DNA in canonical R-loops increases the susceptibility of these regions to nuclease-mediated cleavage, secondary-structure formation, replication-fork collapse, and double-strand breaks. As a result, recruitment of replication protein A (RPA), ATM/ATR-mediated checkpoint activation, and histone H2AX phosphorylation (γH2AX) may establish connections between persistent hybrid-associated structures and genome stress independently of a completed integration event [110]. There is yet another class of molecular alterations generated as a result of producing cDNA through reverse transcription. cDNA produced through reverse transcription can transiently exist without the nucleosomal packaging or epigenetic modifications characteristic of native genomic DNA and may therefore exist outside its usual chromatin-regulatory environment. Ultimately, the disposition of cDNA will depend upon physical properties such as length, orientation, terminal structure, associations with proteins, and subcellular localization [111].

It is worth noting that cDNA produced during target-primed reverse transcription by LINE-1 elements is generated at a chromosomal target site and therefore is expected initially to exist predominantly in the nucleus and to be associated with local genome stress at that site. However, whether some proportion of abortively synthesized cDNA or related intermediate products becomes physically released or otherwise redistributed to cellular compartments in which they could act as innate immune ligands remains unclear and should not be inferred solely from the occurrence of reverse transcription [112].

While productive retrotransposition will ultimately place the newly synthesized sequence into chromatin, thereby placing it again under host chromatin regulation, abortive reverse transcription can generate numerous transient but potentially biologically active products, including DNA nicks, RNA–DNA duplexes, single-stranded DNA termini, and partial-length cDNA. These products can be accessible to genome-maintenance machinery and, where appropriate molecular and spatial conditions are met, may also become accessible to innate immune surveillance machinery [113]. Productive and abortive retrotransposition should therefore be viewed as separate biological endpoints. Productive retrotransposition results in placement of newly synthesized sequences into chromatin, where they again become subject to host regulation. Abortive retrotransposition produces numerous transient but potentially biologically active products, including DNA nicks, RNA–DNA duplexes, single-stranded DNA termini, and partial-length cDNA, that are accessible to genome-maintenance machinery and, when appropriate spatial and molecular conditions are met, may also become accessible to innate immune surveillance machinery. Indeed, many of the transient molecular intermediates resulting from retroelement activity may produce more immediate biological effects than the eventual and much rarer endpoint of complete integration into chromatin [25].

Unlike a linear series of enzyme-catalyzed reactions, the biochemical conversions that occur during retroelement activity are better represented as a branching network. For example, after transcription, an RNA molecule can be degraded; it can form an RNA–RNA duplex; it can undergo completion of RNP maturation; it can form or persist as an RNA–DNA hybrid; it can undergo reverse transcription with subsequent completion or premature termination of cDNA synthesis; or it can eventually integrate stably into chromatin [114]. Each branch represents a unique combination of enzymatic requirements and resultant biological effects. Therefore, experimental attention should focus on determining the rates of transition through each branch or molecular state rather than measuring only overall levels of retroelement RNA [115].

One of the key mechanistic issues is therefore how cells recognize abnormal molecular states produced by aberrant retroelement activity. If structurally competent RNA, RNP-associated products, or reverse-transcribed DNA persist beyond normal processing times, their subsequent biological fate will likely depend upon which surveillance pathway encounters these species, whether restriction occurs before productive signal transduction, and whether such signaling ultimately converts recognition into a broader cellular response [116]. Importantly, continued accumulation of a candidate ligand together with activation of a downstream signaling pathway does not by itself demonstrate direct sensor–ligand engagement. This level of molecular discrimination is further discussed in Section 4.

## 4. Innate Sensor Selection and Pre-Sensor Host Restriction

To be recognized as a ligand (i.e., to become immunologically significant), retroelement-derived nucleic acids must acquire an appropriate physical structure and localization in order to access the relevant innate-sensing pathway. Ligands may lose their ability to interact with receptors due to degradative processes, post-transcriptional modification of nucleic acids (editing), binding by proteins that shield them from interaction with the immune system, sequestration away from relevant sensors, compartmentalization within cellular structures that prevent sensor access, and/or trafficking [117,118]. Sensor identification is therefore dependent on evidence beyond the presence of candidate ligands and subsequent activation of an innate-sensing pathway; preferably, this will include direct detection and/or isolation of receptor-bound ligands, together with determination of whether the identified ligands require specific sequences derived from retroelements, genetic studies demonstrating functional dependence upon these sequences, and separation-of-function analyses [119].

### 4.1. Cooperative Sensing of dsRNA Filaments by MDA5 and RIG-I

Cooperative filament formation by MDA5 results in the creation of filaments assembled along dsRNA. When filaments are formed, the N-terminal CARD domains of receptors associated with the filament are brought into sufficient proximity to facilitate MAVS nucleation. The competence of a molecule to act as a ligand for MDA5 is based upon its ability to form continuous duplex RNA and maintain associated receptors for a sufficient period of time [120]. MDA5 discrimination is kinetic in nature. Relatively slow filament nucleation and ATP-dependent filament-end disassembly favor loss of stable filament formation on both shorter and discontinuous dsRNA species. Conversely, longer dsRNA species can support sustained elongation and repair of filament discontinuities [121]. Although considerable amounts of RNA can be generated within cells, minimal inflammatory signaling may arise from RNA containing single-base mismatches, looped secondary structures, post-transcriptionally edited regions, or protein coatings that prevent effective filament formation. Low-abundance RNA containing long stretches of accessible dsRNA may, in contrast, exhibit greater immunogenic potential. Mapping studies examining impaired self-RNA editing indicate that only a small percentage of total dsRNA contained within cells has sufficient properties to activate MDA5 [122]. Potential substrates for MDA5 filament formation include regions of transcripts that possess extensive secondary structure, including inverted repeats, ERV-derived RNA, and other configurations that produce dsRNA through extensive complementarity. Studies documenting associations or colocalization of MDA5 with repeat-derived RNA further support the idea that certain subsets of dsRNA possess structural features that make them particularly capable of stimulating MDA5 in a virus-like manner; moreover, this stimulation is not due simply to nonspecific recognition of repetitive transcription [123].

ADAR1-induced structural alterations modify the threshold required to activate MDA5. A-to-I editing by ADAR1 creates noncanonical base-pairing environments that disrupt base stacking and reduce duplex continuity. Experiments using defined substrates have demonstrated measurable effects of A-to-I editing on MDA5 filament assembly. A mechanistic relationship between enzymatic modification of dsRNA and supramolecular receptor assembly has thus been demonstrated. Whether a substrate has undergone A-to-I editing is therefore less important than whether sufficient residual duplex RNA remains available for cooperative receptor assembly [124].

MDA5 is distinct from RIG-I in terms of molecular filtering. While compatibility with terminal chemistry can significantly affect RIG-I responsiveness, terminal compatibility alone is insufficient to activate RIG-I. Competitively bound endogenous proteins can restrict access to the terminus of RNA, thereby limiting RIG-I access. For example, certain endogenous proteins selectively bind to the termini of 5′-triphosphorylated duplex RNA, limiting RIG-I access [125].

In addition to the chemical and physical characteristics inherent in the RNA itself, the competence of a ligand also depends upon the quantity and types of proteins attached to its surface [126].

In addition to differences related to sensitivity to long versus short populations of dsRNA, several additional distinctions exist between MDA5 and RIG-I. Much of MDA5 signaling arises from internal duplex continuity and cooperative filament formation. Terminal chemistry and accessible ends represent major determinants of RIG-I activity. Modifications made by ADAR1 can compromise the structural integrity required for MDA5, while competitive binding by endogenous proteins can preclude RIG-I from accessing otherwise compatible ligands [127].

### 4.2. Discrimination Among Z-Nucleic-Acid Geometries by ZBP1

Additional discriminatory capacity for specific geometric arrangements of nucleic acids is provided by ZBP1. ZBP1 recognizes left-handed Z-DNA and Z-RNA through its two N-terminal Zα domains and differs from MDA5 and RIG-I in that it recognizes specific higher-order conformations of nucleic acid rather than relying solely on duplex formation or terminal chemistry. Following recognition of Z-RNA or Z-DNA by ZBP1, RHIM-dependent interactions can recruit RIPK3 and, under certain conditions, promote RIPK3-dependent activation of MLKL-mediated necroptosis or other inflammatory and cell-death pathways [128].

Well-characterized experimental systems demonstrate direct activation of ZBP1 by Z-RNA followed by RIPK3–MLKL signaling [129].

Responses elicited by ZBP1 are highly context-dependent. Studies of impaired ADAR1 function suggest that endogenous complementary retroelement-derived RNAs can provide candidate Z-RNA substrates for ZBP1; however, ZBP1-dependent type I interferon-mediated pathology can develop independently of RIPK1, RIPK3, MLKL, and caspase-8 in some experimental models [19].

Therefore, activation of ZBP1 should not be assumed to be invariably associated with necroptosis [130].

Recent work employing a well-characterized mouse model of tauopathy has documented a potentially relevant link to neurodegeneration. Disruption of heterochromatin organization associated with tau was linked to reactivation of transposable elements, formation of endogenous Z-RNA, and activation of ZBP1 in neurons. Reduction in Zbp1 expression alleviated neurodegenerative phenotypes in this model [131].

These data support the notion that transposable-element-derived Z-RNA can activate ZBP1 in a defined neurodegenerative context but do not imply equivalent dependence on ZBP1 across all neuroinflammatory diseases or in human disease [132].

Because MDA5, RIG-I, cGAS, and ZBP1 recognize different structural and spatial characteristics of nucleic acids, the presence of retroelement-derived RNA or DNA alone is insufficient to assign a specific innate sensor [133]. Table 2 summarizes the principal molecular determinants recognized by these surveillance pathways and the experimental criteria required to distinguish candidate ligands from demonstrated sensor substrates.

### 4.3. cGAS–STING: DNA Sensing and a Proposed Pre-Sensor LINE-1 Restriction Pathway

While cGAS itself does not detect DNA based primarily on where it originates or the original sequence contained in the DNA template, cGAS detects DNA that is sufficiently exposed to support productive cGAS assembly and 2′3′-cGAMP synthesis. Thus, DNA created from a retroelement will only be detected by cGAS if it reaches a molecular and cellular environment that permits cGAS recognition, rather than being degraded, incorporated into chromatin, or protected by proteins prior to detection [134]. It is worth noting that simply demonstrating the existence of LINE-1 reverse transcription or cDNA accumulation provides no direct evidence of cGAS engagement; instead, appropriate compartmentalization, physical sensor association, and/or genetic epistasis are required to identify a particular retroelement-derived DNA species as a cGAS ligand [102].

Evidence from human cells (cell culture) shows that STING associates with LINE-1 ORF1p and that this association is accompanied by the routing of ORF1p-containing material to various cellular compartments, including those involved in endoplasmic reticulum (ER)-associated, Golgi-related, and endolysosomal processes, along with a reduction in ORF1p persistence and LINE-1 retrotransposition [19]. Furthermore, perturbing STING, as well as defined trafficking-related STING amino acid residues, results in impairment of these processes, thus supporting a cGAS-independent restriction function that occurs prior to extensive reverse transcription. Therefore, the data presented support the notion that STING participates in ORF1p cargo processing; however, they do not provide evidence that STING itself is capable of transporting ORF1p and further do not demonstrate the quantitative importance of this pathway in either neurons or microglial cells in vivo. The specific sorting apparatus utilized by this pathway, as well as the physiological significance of this mechanism within the central nervous system, will have to be assessed directly [135].

The action performed by STING in this context is separable from its canonical signaling role. Efficient routing of ORF1p-containing material toward lysosomes and repression of LINE-1 retrotransposition require the acidic residue pair E282/D283. Mutation of these residues impairs lysosomal targeting and makes repression of ORF1p less effective than with wild-type STING. Another pair of acidic residues, E296/D297, is not required for STING-associated routing of ORF1p-containing material and thus provides evidence that STING-mediated transport of ORF1p involves a localized transport determinant rather than an indirect effect due solely to acidic charge. Moreover, dimerization of STING appears to be necessary for retroelement cargo processing, indicating that organization of STING dimers plays an important role in processing retroelement cargo [19].

As noted above, this pathway of degradation does not correspond directly to conventional macroautophagy. Loss or disruption of key proteins involved in the autophagic pathway, including ATG5, ULK1, ATG9A, p62, and Beclin1, does not prevent STING-mediated repression of ORF1p. Likewise, disruption of the ESCRT-associated proteins TSG101 and ALIX does not completely abolish STING-mediated repression of ORF1p. Overall, the data indicate the presence of a distinct membrane-trafficking pathway with a poorly characterized sorting apparatus. Other vesicular pathways, including Golgi-to-endosome/endolysosome sorting, as well as other mechanisms involving lysosomal internalization of cargo, therefore remain viable alternatives. Nonetheless, no specific microautophagic or alternative trafficking pathway has yet been definitively established [136].

Hence, we may conceptualize the relationships among these elements as being inverted relative to what would normally occur in a typical nucleic-acid-sensing process:

STING → elimination of ORF1p → reduction in reverse transcription → reduction in formation of aberrant DNA

can precede

retroelement-derived DNA → cGAS → 2′3′-cGAMP → STING

thereby permitting STING to perform roles in both pre-ligand-formation and post-ligand-recognition contexts. This duality is mechanistically important because pre-sensor restriction acts against the source of aberrant DNA rather than continuously reacting to its products. Currently available evidence does not demonstrate competition between cargo processing and canonical signaling for a limited pool of STING molecules [137].

Likewise, there is considerable interconnectivity among the pathways described above. Recent studies indicate that human cGAS controls LINE-1 transcription through mechanisms involving CTCF- and RUNX3-associated transcriptional regulation, leading to elevated levels of LINE-1 RNA that may subsequently contribute to MAVS-dependent cellular responses [138,139]. This relationship was found to differ between human and mouse systems; therefore, it is not valid to assume that this regulatory pathway will function identically across species or in microglia. Nonetheless, it demonstrates that innate pathways can affect each other’s potential ligand pools rather than functioning independently as terminal endpoints [140].

### 4.4. Type I Interferon Feedback: Will Detection Allow Restoration of Containment or Facilitate Entry into Persistent States?

One way in which the choice between restoring molecular control or entering a persistent state following sensor activation may be influenced is through activation of type I interferon (IFN-I) signaling. Activation of type I interferon signaling activates JAK1 and TYK2, promoting formation of STAT1–STAT2–IRF9 complexes and leading to various forms of antiviral activity, including RNA-editing enzymes, nucleases, helicases, restriction factors, and other pattern-recognition machinery. Accumulation of repeat-control components, including DAXX, can also occur during IFN-responsive states, potentially establishing a feedback loop in which recognition of escaped material enhances the ability to restrict future escape [141].

Therefore, type I interferon feedback could act as a homeostatic feedback mechanism:

retroelement-derived ligand → innate sensor → IFN-I → induction of restriction-factor gene expression → decreased persistence of ligand

Nonetheless, there are limits to this feedback mechanism. Disruption of IFNAR signaling can suppress IFN-stimulated transcription and thereby restore aspects of homeostatic function in microglia. However, programs associated with ongoing DNA damage, cell-cycle dysregulation, p53/p21 signaling, and disease-related cell states may continue to persist and retain characteristics indicative of a damaged cell state. Activated reactive programs can also remain active even after suppression of IFN-mediated transcriptional responses. Together, these observations suggest that there are at least two partially discrete components of the phenotype: an IFN-dependent antiviral phenotype and an IFN-independent state transition caused by an underlying genomic or cellular defect [142].

Therefore, type I interferon production should not be universally considered the primary pathology. Rather, in certain situations, it may represent an attempt to correct existing defects through compensatory programs initiated by continued production of ligands or genome stress. Blocking IFN can diminish one transcriptional program without diminishing the underlying cause of the lesion [143].

A similar duality is seen for STING. Chronic inflammatory transcription can result from sustained cGAS–STING signaling; however, STING also contributes to maintaining genomic and tissue homeostasis throughout the aging CNS through its actions in microglia. For instance, loss of STING has been associated with increased DNA damage and altered gene-expression profiles in microglia, compromised blood–brain barrier integrity, and worsened neurological dysfunction in defined models of aging. Therefore, the biological consequences generated by STING depend on which component predominates: retroelement quality control; acute cGAMP surveillance; membrane-associated stress responses; or chronic downstream inflammatory transcriptional programming [144].

It is therefore useful to conceptualize the system as having three parameters: ligand creation; ligand visibility; and restriction capacity. Retroelements produce the initial input; modifications, including enzymatic degradation, protein shielding, and localization, determine how much of that input is observable; MDA5, RIG-I, cGAS, and ZBP1 convert structurally compatible observable inputs into signals; STING and MAVS convey these signals, while STING can also participate in processing the sources of those inputs; finally, type I interferon feedback attempts to increase the threshold at which further escape will be tolerated [145]. If the sum total of these processes is capable of maintaining ligand flux below the level that effective restriction capacity can accommodate, homeostasis can be maintained. If ligand flux continues to exceed restriction capacity, persistent innate activation and unresolved genome stress may coexist. At this time, this flux-over-capacity relationship should be viewed as a mechanistic framework rather than a demonstrated universal quantitative threshold [146].

These interactions indicate that LINE-1 surveillance cannot be reduced to a single sensor or signal-transduction event but instead relies on sequential checkpoints regulating cargo disposal, reverse transcription, DNA visibility, sensor activation, and interferon-mediated restriction [147]. Table 3 summarizes these checkpoints and distinguishes successful containment from persistent innate activation when ligand generation exceeds effective restriction capacity.

## 5. Persistent Microglial-State Remodeling: A Working Model of Molecular Hysteresis

Microglia that have been exposed to persistent levels of either cellular or genomic stress following chronic stress can remain altered for extended periods, and while these changes suggest that there may potentially be hysteresis, they provide no definitive proof of it. Hysteresis will be defined here as a proposed and experimentally testable paradigm for describing path-dependent persistence, whereby an altered cellular state (microglia) persists after both verification that the original perturbation has been removed or normalized and evidence of a non-equivalent reversal trajectory. Therefore, the mechanisms described below are potential contributing factors to this persistence but should not be considered as having been proven to create a “state-fixation” pathway [161,162].

Recently, an important research study investigated how DAXX-based chromatin regulation influences the presence of both endogenous retroelements and persistent microglial phenotypes. The researchers used tamoxifen-inducible Cre recombinase under the control of the Cx3cr1 promoter, along with the Daxx fl/fl allele, to create mice lacking Daxx in their adult microglia [163]. In addition to examining ex vivo cultured cells from these mice, Yan et al. demonstrated that loss of Daxx resulted in lower levels of H3K9me3 at several long terminal repeat (LTR) retroelements, while H3.3 remained abundant at most sites. Loss of Daxx was also associated with rapid derepression of retrotransposons, altered transcriptional patterns, and re-entry into the cell cycle [164]. These events were subsequently associated with increased evidence of DNA damage, altered numbers of microglia, and populations of Apoe-high, lysosome-containing microglia that exhibited multiple features of cellular senescence. Deletion of Pml in combination with Daxx deficiency altered some aspects of the senescent phenotype but did not restore all aspects of the reactive microglial phenotype. Therefore, PML may play a role in maintaining specific downstream traits resulting from the initial reactive microglial response rather than being the primary cause of prolonged microglial dysfunction [165].

### 5.1. DNA-Damage Checkpoints as Candidate Drivers of Persistent Cell-State Transitions

Rather than describing the heterogeneity and complexity of microglial responses to genome-wide stress using a single indicator representative of a particular cell state, such as senescence, the individual phases of the microglial response can be better represented as a sequence of checkpoint activations [166]. Each checkpoint offers different choices depending on where the cell is positioned spatially and temporally. For example, when p53 activation and γH2AX formation occur simultaneously, several possible outcomes can arise, including continued proliferation, programmed cell death, and prolonged cessation of growth. Pro-apoptotic BAX/BAK and antiproliferative CDKN1A/p21 programs can coexist with signs of ongoing cell division, as shown by Ki-67-positive staining. Thus, stressed microglia occupy a multidimensional decision space that can yield several possible outcomes in response to different exposures to stressors [167].

Decisions made by checkpoints are partially mediated through p53. If DNA damage remains compatible with viable cell function, then CDKN1A, or p21, may be induced. Induction of p21 results in inhibition of CDK2 and CDK4/6 kinase activity, maintenance of hypophosphorylated retinoblastoma protein (RB), and inhibition of E2F-dependent progression through the G1–S transition [168]. However, greater quantities or longer durations of DNA damage can also induce mitochondrial apoptosis through BAX/BAK. Therefore, these two forms of output are not necessarily mutually exclusive across all members of a population of cells. This provides a rationale for why proliferation, cell death, and subsequent arrest can occur sequentially along the same pathological pathway [169].

The immediate proliferation observed following genome-wide stress illustrates this point particularly clearly. Microglia exposed to genome-wide stress have been found to express high levels of Ki-67 immediately after stress, followed by divergence toward either restoration of normal homeostatic characteristics or persistence in APOE-high, damage-associated phenotypes. Therefore, entry into the cell cycle following DNA damage is not merely inversely related to senescence; rather, it may signify that the cell is attempting to divide despite incomplete repair of the underlying defect. Similarly, entry into S phase during ongoing DNA damage exposes unresolved lesions to replication-fork-associated stress, enhances checkpoint signaling, and may increase the probability that an initially transient response evolves into a persistent response [170].

Consequently, we distinguish between damage generation and damage memory. Damage generation is reflected by markers such as γH2AX and activated p53. Candidate features of damage memory could include prolonged p21 expression, limitations imposed on E2F/RB-mediated cell-cycle progression, and subsequent reorganization of nuclear components. Upon achieving sufficient stability among checkpoint mechanisms to encode previous stress exposure as part of the cellular state, withdrawal of the original inflammatory stimulus may reduce the intensity of the acute transcriptional response without restoring proliferative capacity or the pre-existing condition of the microglial cells. Therefore, if prolonged operation of checkpoint mechanisms develops partial independence from the original insult and depends on the route through which the altered state was achieved, evidence supporting hysteresis would emerge [171].

### 5.2. PML Nuclear Bodies as Candidate Contributors to Persistent Nuclear-State Organization

To date, numerous findings suggest that PML may serve as a bridge between checkpoint signaling and spatial organization of nuclear memory. Unlike traditional senescence indicators, PML acts as a TRIM-family scaffold. PML self-assembly into oligomeric structures, together with SUMOylation-dependent recruitment, organizes DNA-damage-responsive and transcriptional regulators within distinct subnuclear compartments referred to as PML nuclear bodies (PML-NBs) [172].

Structural studies have allowed characterization of important features underlying PML-NB organization. The α-helical framework of the PML RBCC region is supported by hydrophobic residues including F108, F109, L112, and L116. The dimerization interface of PML includes V123, F177, and L178. Mutations affecting F177 can substantially impair PML-NB formation, whereas mutations affecting V123 or L178 can modify the number or size of PML nuclear bodies. Higher-order oligomeric assemblies extending from the dimeric core allow larger nuclear structures to form. In addition, surface-exposed residues K65 and K160 remain available for SUMOylation, allowing recruitment of SUMO-interacting protein partners after formation of the PML scaffold [173,174].

The creation of higher-order assemblies by PML may enhance the persistence of molecular interactions related to checkpoint signaling. Formation of these assemblies results in increased local concentration and longer residence times of SUMOylated p53-regulatory proteins, DNA-repair proteins, and DAXX within multivalent structures. This may result in prolonged association of factors whose soluble concentrations decrease rapidly when their initiating stimuli are removed, thereby providing additional opportunities for molecular interaction and modification [175].

PML-NBs can therefore be considered dynamic, multivalent nuclear compartments exhibiting condensate-like properties. However, there is insufficient evidence to assume that PML-NB organization is equivalent to canonical liquid–liquid phase separation (LLPS), and the contribution of LLPS to PML-NB behavior in microglia remains unresolved [176].

Studies utilizing genetic deletion have provided support for this view. Genetic deletion of PML in DAXX-deficient microglia resulted in a significant decrease in features associated with senescence but did not correct abnormal morphology, elevated CD68 levels, or abnormal proliferation exhibited by DAXX-deficient microglia. Therefore, PML does not appear to be essential for generating the primary injury or the broader reactive phenotype that occurs in response to injury, but it appears to participate in the maintenance of particular downstream states. Elimination of an arrest program dependent on PML does not appear to be equivalent to restoration of homeostasis [177,178].

Therefore, a distinction exists between withdrawal from an arrest program and restoration of homeostasis. Once genome-wide stress responses have developed, p53 has been activated, sustained p21 expression has occurred, and PML bodies have formed, cells may not follow the same molecular trajectory once the initiating insult is eliminated. Therefore, restoration of chromatin integrity in regions affected during the initial injury may not suffice to restore the pre-injury cellular state if subsequent nuclear configurations develop a degree of functional autonomy. Whether a true PML-associated microglial hysteretic phenomenon exists will ultimately depend upon experimental demonstration of path dependence rather than persistence alone [179].

### 5.3. APOE-High, Lysosomal and Metabolic Remodeling as Candidate Contributors to Persistent Microglial States

Persistent microglial states can also have a number of profound effects on cellular functions such as lipid transport, lysosomal function, and intermediary metabolism. Microglia can transition from homeostatic states associated with SALL1/P2RY12/CX3CR1 to APOE-high, lysosome-rich states. The degree to which these mechanisms contribute to long-term microglial persistence is uncertain and will likely depend upon the type of initial insult and the experimental conditions. Lysosomal and metabolic modifications are therefore considered examples of potential mechanisms that may contribute to the maintenance of microglial persistence, rather than as evidence that microglial persistence is fixed in some form [180].

APOE has served as a molecular indicator of where cells have departed from their normal trajectory of homeostasis. Since APOE-positive, LAMP1-positive structures and increased programs of vesicular trafficking occur together with dystrophic neurites in regions where cellular homeostasis has undergone prolonged deviation from its normal state, accumulation of APOE together with alterations in lipid and lysosomal function may become incorporated into persistent transcriptional profiles of microglia rather than representing simply secondary consequences of tissue injury [181].

There is substantial similarity in the downstream architectural characteristics defining dysfunctional microglia among human microglial models displaying disruption of mitochondrial proteostasis. These similarities include elevated levels of γH2AX, p16, and p21; increased inflammatory mediators; altered NAD^+^ metabolism; PARP1-dependent cellular stress; and significant alterations in methionine, folate, transsulfuration, and lipid metabolism. The fact that different forms of mitochondrial proteostasis disruption can produce a similar microglial state does not imply that mitochondrial proteotoxicity is analogous to retroelement biology. Rather, it suggests that disruptions of different upstream biological processes can converge upon a common, attractor-like microglial state that includes genome stress, proliferative arrest, lysosomal dysfunction, and chronic inflammation [182].

Altered methionine, S-adenosylmethionine, and polyamine synthesis may be part of the mechanism through which certain characteristics of the microglial cell state persist under conditions of chronic stress. While alterations to MAT2A or reductions in spermidine levels can sometimes reduce subsets of p21, DNA-damage, and/or inflammatory readouts, such changes will usually fail to reverse the initiating stress-signaling pathway. Therefore, while these findings support an important contribution of secondary metabolic remodeling to chronic stress responses, they do not support the conclusion that the pathways studied here are necessary for sustaining the same states [183].

There are many reasons why microglia may remain dysfunctional for an extended period. Neutral-lipid remodeling is one potential contributor. When defined experimental paradigms are used to modify DGAT1/2-dependent triacylglycerol storage, this alters the levels of γH2AX, p21, and inflammatory readouts. Therefore, there is evidence that lipid accumulation functions in conjunction with other aspects of the altered cellular phenotype, rather than simply being indicative of increased activation. It remains to be determined whether this relationship is necessary, sufficient, or preserved across different types of microglial states and neurological diseases [184].

Evidence consistent with hysteretic phenomena can be obtained by comparing acute versus chronic insults. Antioxidant therapies may decrease DNA damage and p21 production during acute stress but can lose efficacy after chronic establishment of the initiating lesion. Conversely, partial reduction in senescence-related gene-product expression can be achieved through manipulation of SAM, polyamine, or neutral-lipid metabolism without correcting deficits in mitochondrial proteostasis [185].

Separation between initiation and maintenance supports the existence of distinct mechanisms underlying these phenomena. However, formal demonstration of hysteresis will require trigger-removal experiments together with evidence of path dependence [186].

**Figure 2:**
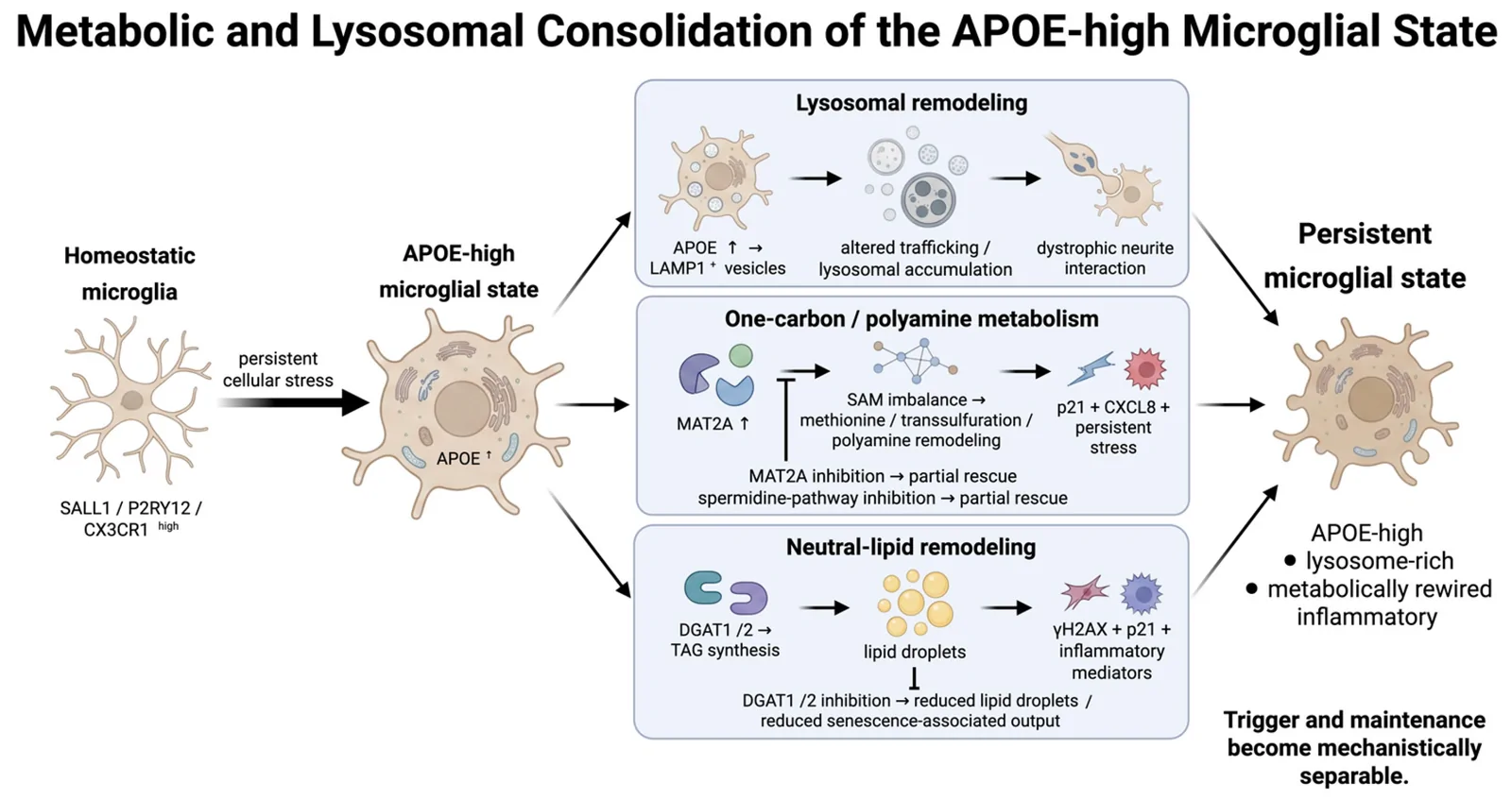
Metabolic–lysosomal consolidation of the APOE-high microglial state. Persistent stress promotes transition from homeostatic microglia toward an APOE-high, lysosome-enriched phenotype. Convergent metabolic programs involving MAT2A/SAM–polyamine remodeling and DGAT1/2-dependent lipid storage reinforce p21, γH2AX and inflammatory outputs. Their partial reversibility despite persistent upstream stress supports a distinction between state initiation and state maintenance.

Therefore, trigger and maintenance appear to be mechanistically separable. A model emerges in which multiple forms of molecular memory may interact to maintain chronic dysfunction in microglia, including: (1) checkpoint memory via p53–p21–RB circuitry; (2) structural memory via PML nuclear bodies; (3) transcriptional memory via persistent use of regulatory elements; (4) organelle memory via lysosomal remodeling; and (5) metabolic memory via alterations in SAM, polyamine, and lipid fluxes. Of course, there is no requirement that any of these forms of memory be individually irreversible. Their combined effect may nevertheless result in a condition in which restoration requires more than removal of the initiating insult [187]. A diagrammatic representation summarizing the convergence of lysosomal, one-carbon/polyamine, and neutral-lipid remodeling toward an APOE-high microglial state and the potential uncoupling of maintenance from the initiating insult is provided in Figure 2.

Ultimately, an important consequence is that a cell-intrinsic defect can potentially acquire a partially autonomous downstream logic. When nuclear, lysosomal, and metabolic programs begin to mutually reinforce one another, the altered microglial phenotype may selectively affect neighboring neurons through altered phagocytic activity, lipid exchange, or complement competence [187]. Section 6 therefore addresses what happens next: how intracellular molecular history is translated into selective synaptic and circuit pathology.

## 6. Intercellular Conversion: From Epigenetic Failure to Synaptic Pathology

The conversion of malfunctioning cells into malfunctioning networks requires some form of conversion mechanism for transforming the intracellular defects of cells, i.e., their molecular history, into an intercellular signal. The complement system has a conversion mechanism for this type of transformation because it can convert the cellular stress that a neuron undergoes into an intercellular signal and altered microglial behavior, while simultaneously altering the trafficking of membrane proteins that are internalized within a neuron [188]. Thus, as a result of this evidence, investigators are beginning to question the idea of a “comprehensive theory of complement-mediated pruning.” Depending on the various elements of the complement system, the subcellular compartment of a neuron, and the condition of a neural network, the complement system can serve as an extracellular labeling system through opsonization, alter microglial integrin signaling, alter the dynamic properties of microglial processes, or alter intracellular endosomal trafficking. The key issue here is that the conversion of complement-associated information into functional synaptic dysregulation is not likely to depend simply on whether or not the complement system is activated [189].

### 6.1. ERV-Derived RNA–DNA Hybrids Couple Neuronal SETDB1 Loss to Complement Competence

Recently, a study demonstrated a pathway connecting the neuronal epigenome to the complement system in prefrontal cortical neurons. Loss of SETDB1-mediated repression and/or maternal inflammatory exposure resulted in re-expression of ERVs, accumulation of ERV-derived RNA–DNA hybrids, and increased C4b in neurons. Most importantly, the order in which these events occurred is mechanistically important, as both the initial loss of repression and the subsequent complement response occurred in neurons; however, microglia served as effector cells downstream of the neuronal lesion. Overall, activation of the complement system can occur in conjunction with specific intracellular nucleic-acid states and does not require generalized inflammation-induced tissue damage [28,65].

Reverse transcription appears to play a role in this process. Treatment with drugs that inhibit reverse transcription reduced the amount of RNA–DNA hybrids, reduced neuronal C4b, and attenuated synaptic abnormalities as well as microglial and behavioral abnormalities. These data suggest that transcription of ERVs alone cannot explain the observed phenotypes. A more accurate representation of the relationship described above is:

SETDB1 deficiency → ERV-derived reverse-transcription intermediates → induction of neuronal C4b → microglial synaptic remodeling

Genetic and biochemical studies support the sequence of events described above. Specifically, removal of microglia improved synaptic pathology regardless of whether the neuronal epigenetic lesion was corrected. This indicates that microglia are positioned downstream of neuronal complement signaling. Furthermore, deletion of C4b rescued synaptic and behavioral abnormalities. This indicates that the complement system is positioned between the retroelement-derived intermediate and microglial execution. Finally, treatment with drugs that inhibit reverse transcription further supports a causal role for reverse transcription at an earlier stage by reducing the amount of molecular intermediates associated with the complement-related phenotype [190].

Thus, the remaining question involves identifying the biochemical mechanism or mechanisms by which RNA–DNA hybrid accumulation leads to C4b induction. It is possible that several biochemical mechanisms can link an RNA–DNA hybrid to C4b induction, including:

(a) An RNA–DNA hybrid could represent a stable change in either the properties of a DNA region during transcriptional elongation or its topology [107];
(b) An RNA–DNA hybrid generated in cis could recruit DNA-damage-repair machinery or chromatin remodelers [191];
(c) Processing of an RNA–DNA hybrid could generate secondary nucleic-acid species that activate one or more signaling pathways leading to expression of C4b [109].

Depending on their physical and spatial locations, different biochemical mechanisms coupling an RNA–DNA hybrid to C4b induction may therefore be primarily cis-regulatory, genome-stress-related, or signaling-dependent [192].

This pathway provides yet another paradigm: productive insertion into the genome is not necessary for retroelements to participate in disease pathogenesis. Intermediate forms of nucleic acids may be sufficient to modify neuronal complement competence so that an intracellular genomic lesion can be converted into an intercellular synaptic signal [193].

### 6.2. Complement Operates Through Separable Phagocytic, Trafficking and Contact-Regulatory Mechanisms

Traditionally, it has been assumed that the complement system functions in a relatively straightforward manner. Following cleavage of C3, C3 fragments, particularly iC3b, can decorate synaptic material and engage CR3 (αMβ2; composed of CD11b and CD18) on microglia, creating a linkage between the labeled synaptic element and cytoskeletal rearrangements that enable phagocytic removal of labeled synaptic material into lysosome-containing vesicles. Therefore, in addition to increasing the probability of phagocytic activity at a synapse, the complement system can facilitate phagocytic removal of labeled synaptic material [194].

However, evidence now exists indicating that this pathway can be region specific within the cerebral cortex. Chronic stress produces selective deposition of C3 in superficial layers of the medial prefrontal cortex, along with increased microglial CD68 staining, indicating increased lysosomal content, and increased ingestion of VGLUT2-positive terminal segments. Elimination of C3 prevents the associated increase in synaptic uptake, preserves synaptic density within the affected area, and diminishes development of the associated APOE-high microglial phenotype. In this context, C3 serves both as an opsonic marker and as a determinant of the local microglial effector response [195].

In addition, the initial proteolytic pathway may not follow what would be expected from the traditional developmental cascade. High levels of C3 deposition can occur without commensurate levels of C1q deposition; thus, it is difficult to infer that the particular pathway mediating local C3 activation corresponds to classical-pathway activation. Enzymatic pathways generating activated forms of C3 should therefore be investigated directly rather than inferred from the spatial distribution of C1q deposition [196].

Alternatively, high levels of C4 can impede communication among cortico-cortical circuits through an entirely distinct intracellular mechanism involving modulation of endosomal sorting processes that recycle membrane cargo, such as GluA1-containing AMPARs, from endosomes to postsynaptic membranes. C4 and SNX27 colocalize in dendrites and can be recovered together as part of molecular complexes; reducing levels of SNX27 mimics the effect caused by elevated levels of C4 in disrupting synaptic function; conversely, increasing levels of SNX27 restores synaptic function disrupted by elevated levels of C4 [197].

The primary defect in this instance arises from disruption of receptor trafficking rather than diminished production of receptors. Elevated levels of C4 direct GluA1 away from productive recycling pathways mediated by Rab11-positive endosomes toward LAMP1-positive endolysosomal compartments. Consequently, GluA1-containing AMPARs are present at lower levels at postsynaptic membranes although there is no corresponding proportional reduction in the total receptor population. Recycling of AMPARs contributes to maintenance and stability of excitatory synapses through activity-dependent mechanisms. Therefore, disruptions in trafficking prior to apparent structural loss of spines may also contribute to synaptic instability [198].

Therefore, a proposed sequence for this paradigm is:

Increased levels of C4 in neurons → defective sorting via SNX27 → deficient recycling of GluA1 via Rab11 → diversion of GluA1 from recycling routes into endolysosomes → reduced availability of GluA1-containing AMPARs at postsynaptic membranes → spine instability

However, there is considerable contextual dependence of complement biology beyond simply distinguishing between C3 and C4. Use of another model in which frontal-cortex layer II/III pyramidal neurons express high levels of C4 causes both excitatory and inhibitory synaptic-transmission abnormalities as well as decreases in dendritic spine density. Both abnormalities exhibit strong dependence on CR3. Overexpression of C4 also results in decreased microglial process motility, decreased volume of tissue surveyed by microglial processes, and fewer contacts made by microglial processes with dendritic spines, shafts, and presynaptic terminals. CR3 deficiency restores these phenotypes. Further, elevated levels of active RhoA accompany these phenotypes, establishing a link between CR3-dependent complement signaling and the actomyosin machinery controlling microglial process dynamics [199].

Importantly, these CR3-dependent changes can occur independently of measurable increases in bulk ingestion of synaptic material. Therefore, CR3 may control connectivity through modifications in the frequency, duration, or quality of interactions between microglia and synapses rather than solely through increases in phagocytic potential. Therefore, dysfunction in synaptic connectivity resulting from complement activity may involve at least three distinct pathways:

C3/iC3b → CR3 → binding and lysosomal degradation of labeled synaptic material

C4 → defective SNX27/Rab11 trafficking → AMPAR missorting → postsynaptic destabilization

C4 elevation → CR3-dependent microglial signaling → RhoA activation → altered surveillance and dynamic interactions between microglia and synapses

As noted previously, quantitatively determining the relative contributions of these mechanisms remains unresolved and will likely vary depending on age, subregion, the pathway through which complement activation occurs, the local processing state of complement components, and local circuit architecture.

Consequently, manipulation of neuronal C4 and/or microglial CR3 interrogates different steps within a transcellular cascade; neither should be considered representative of the predominant mechanism across all experimental models or paradigms. Therefore, “pruning” induced by the complement system should not be viewed as a sufficiently precise mechanistic outcome until opsonization status, receptor dependence, alterations in trafficking, dynamics of microglia–synapse interactions, and actual phagocytic activity have been separately evaluated experimentally [200].

From the perspective of experimental interventions, blocking microglial CR3 and modulating neuronal C4 both occur at different points along the transcellular cascade, each presenting its own particular experimental challenges. With respect to expression on the cell surface, and therefore accessibility to pharmacological or antibody blockade, CR3 on the surface of microglia represents an effector receptor that may provide greater experimental tractability than selectively downregulating C4 on the neuronal side; the latter would likely necessitate genetically or RNA-based strategies targeted to neurons, as well as consideration of the physiological role of the complement system [201]. Modulation of neuronal C4 appears to represent a point that is more “upstream” of CR3-dependent effector responses to injury or pathology; this upstream position could provide a greater degree of mechanistic specificity if it can be demonstrated that neuronal C4 plays a role in the pathophysiology. Furthermore, modulation of CR3 may also interfere with normal microglial surveillance functions, such as adhesion and phagocytosis. Therefore, we view the apparent experimental tractability of blocking CR3 versus the more upstream positioning of neuronal C4 modulation as two distinct, experimentally testable preclinical hypotheses; they are not yet established therapeutic conclusions. A head-to-head comparison of the efficacy and safety of these interventions under identical conditions (e.g., timing of treatment initiation, specificity of action to the affected cell type, and degree of target engagement) will ultimately be required to determine which method provides greater efficacy and/or lower toxicity in a given disease state [202,203].

**Figure 3:**
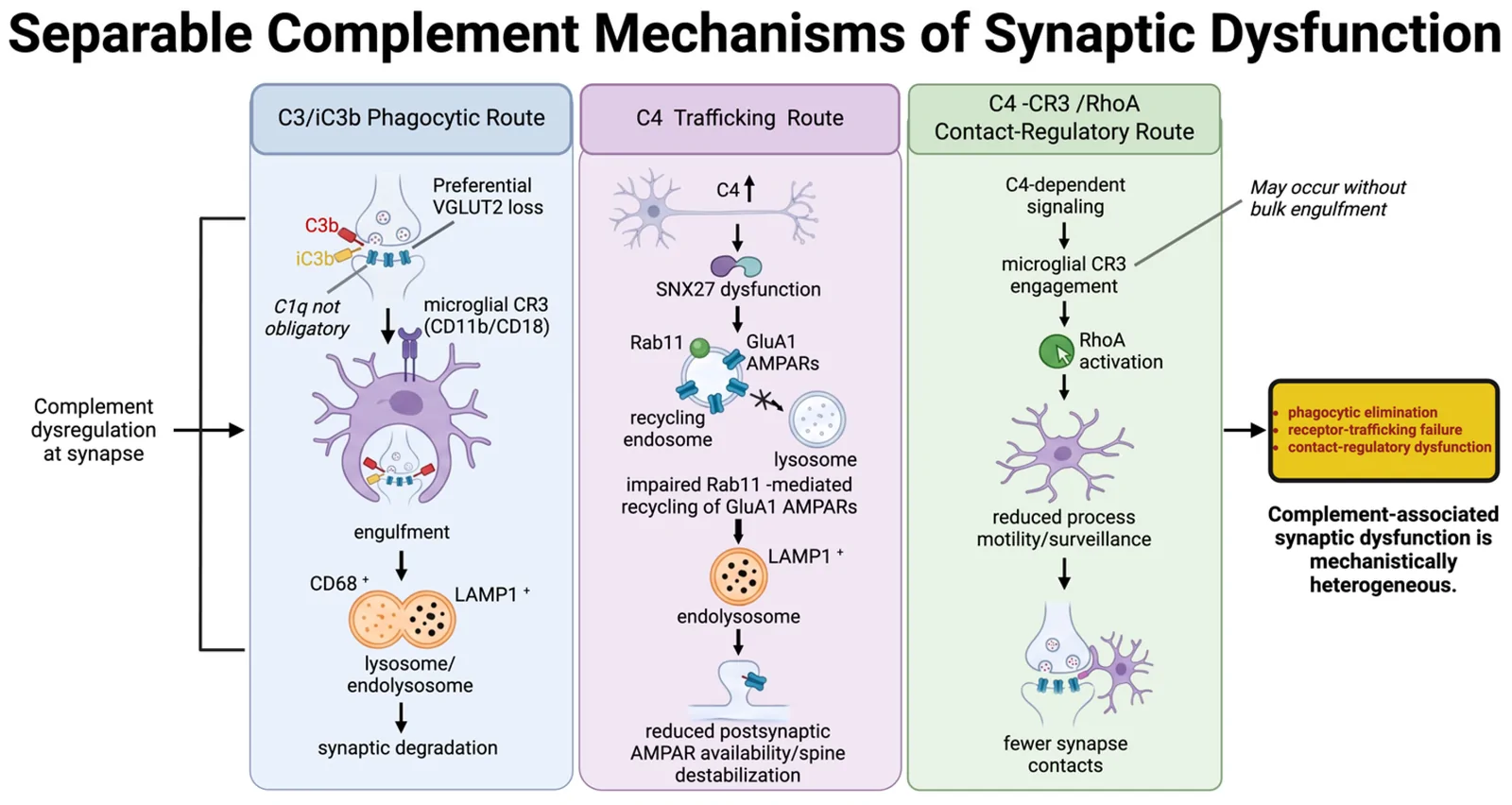
Separable complement mechanisms of synaptic dysfunction. C3/iC3b tagging can promote CR3-dependent microglial engulfment and lysosomal degradation, whereas elevated neuronal C4 can disrupt SNX27–Rab11-dependent GluA1 recycling and reduce postsynaptic AMPAR availability. In parallel, C4-associated phenotypes can require CR3–RhoA signaling to alter microglial process motility and synapse-contact dynamics without increased bulk engulfment. These pathways are separable, context-dependent and not mutually exclusive.

A schematic overview is shown in Figure 3, illustrating how complement can perturb synaptic integrity through mechanistically distinct phagocytic, trafficking and contact-regulatory routes rather than a single pruning pathway.

### 6.3. Circuit Selectivity as a Proposed Model of Serial Molecular Coincidence

The fact that the aforementioned forms of neuroimmune damage occur in a wide range of neurological disorders and disease states and can cause considerable localized disruption to individual synapses does not mean that the spatially localized damage is caused by varying amounts of a single pathogenic molecule. Rather, the damage may occur as a consequence of serial molecular coincidence among the factors responsible for producing the damage [204].

C3 deposition was significantly higher in layers II/III of the medial prefrontal cortex (mPFC) in numerous chronic-stress models than in deeper layers of the cortex. In addition to C3 deposition in layers II/III, several additional effects, including expanded lysosomes in microglia, increased ingestion of VGLUT2-positive material by microglia, fewer VGLUT2/PSD95-positive contacts, more APOE-high microglia, and decreased neuronal recruitment, were observed in layers II/III. All of these events occurred concurrently and were associated with C3 deposition in layers II/III. Most notably, elimination of C3 removed nearly all of these concurrent changes, thereby suggesting that a specific sequence of molecular information moved from the site of complement deposition, through altered microglial function, and ultimately resulted in impaired connectivity [205].

There is a level of spatial specificity below laminar organization. More specifically, layers II/III contain VGLUT2-positive terminals that appear to be primarily affected, while the vast majority of VGLUT1-positive excitatory terminals and VGAT-positive inhibitory terminals appear to be preserved within the same region. Therefore, it would appear that the affected unit is not simply the excitatory synapse but rather a distinct type of excitatory terminal located in a specific anatomical context [206].

The degree of spatial specificity suggests that complement acts at the interface between two cell membranes. The characteristics of cell membranes, the degree of neuronal activity at the synapse, the quantity and quality of extracellular matrix at the synapse, the presence of proteins that regulate complement activation, the amount and topological arrangement of activating ligands, and the structural conformation of complement receptors on the surface of microglial cells at the nanometer scale can determine whether complement components become retained on the target synapse or recognized by microglial processes. Furthermore, CR3 activation state and avidity are also influenced by cytoskeletal integrity. Therefore, opsonization represents an interfacial reaction between a microglial cell membrane and a synapse in which both membranes must be competent to participate in the reaction [207].

Complement activation has additional regulatory steps at the local level. Soluble and membrane-bound regulatory proteins, including CD46, CD55, CD59, and factor H, can modulate the extent and duration of complement activation, convertase formation and stability, persistence of opsonins, and terminal-pathway activity depending upon cell type and species. The contribution of each regulator toward protection against persistent local activation within defined neuronal–glial–synaptic compartments remains incompletely understood. Therefore, persistence of local complement activity depends not only on generation of complement components but also on whether local protective regulation is sufficient to limit their persistence or subsequent recognition by microglial cells [208].

Moreover, another layer of control exists when examining which cellular populations generate complement within brain tissue. While microglia represent an important cellular population potentially contributing to complement production in small areas of brain tissue, complement deposition can remain localized even if microglia are not the primary source of C3 within a given region. Other cell types, including neurons, astrocytes, and endothelial cells, can create independent localized gradients of complement-protein production regardless of the distribution of microglial cells. Therefore, pathology associated with C4 may depend on whether a particular signal remains intracellular, affecting SNX27-mediated transport, or is processed extracellularly and subsequently acts through CR3-dependent pathways in microglial processes [209].

Therefore, susceptibility to neural-network injury may be envisioned as a molecular “AND” gate. At least five separate conditions may need to coexist for pathology to develop: (i) an intracellular lesion that initially produces a complement-inducing signal; (ii) availability of the necessary effectors required to produce pathology; (iii) insufficiency of local protective mechanisms, including complement regulation; (iv) susceptible elements within either synaptic trafficking or membrane states; and (v) available competent microglial cells capable of responding. Elimination of any one condition may prevent development of pathology despite continued presence of the initiating pathological event [210].

Processing in series can provide insight into how a general genome-wide defect results in highly localized circuit dysfunction. Not all cells capable of generating intermediates derived from retroelements are able to convert them into a functional complement-competent state; similarly, only a limited number of cells capable of converting intermediates into a functional complement-competent state have the capacity to activate the appropriate effector mechanisms; further, activation of those effector mechanisms is constrained by local proteolytic processing, complement regulation, receptor topology, synaptic identity, and the local microglial environment. Finally, increasing levels of spatial resolution are created at each stage during which pathological information is transformed from one form into another [211].

Ultimately, neuroimmune injury should be considered an orderly succession of transcellular molecular transformations rather than nonselective passive diffusion of inflammation. Nucleic-acid intermediates are formed following alterations in chromatin; those nucleic-acid intermediates can be converted into defined complement signals; those signals can be used as tags for recognition by microglial cells through opsonization, can induce alterations in intracellular trafficking within neurons, or can signal through microglial surface receptors such as CR3; finally, these transformations can lead to loss or instability of specific synaptic connections. Thus, identification of early reversible transformation points along this cascade becomes experimentally important rather than focusing exclusively on suppression of the eventual inflammatory response [212].

## 7. Causal Interception: A Proposed Experimental Framework for Retroelement Neuroimmunology

To determine the causal ordering among relationships involving the co-occurrence of retroelement derepression, innate signaling, and neurological dysfunction, we have developed an experimental hierarchy that allows for detailed examination of the various components of these relationships. Within this hierarchical structure, each component (genomic locus, molecular intermediate, restriction/sensor pathway, long-term cellular state, intercellular effector(s), and circuit phenotype) could be examined separately through the following experimental methodologies: (1) perturbation, (2) rescue experiments, (3) temporal reversal experiments, (4) separation-of-function testing, and (5) orthogonal measurement. The purpose of this hierarchical approach is to establish a possible framework for future experimentation that provides greater resolution regarding causality and does not represent the standard methodology for the field [213].

### 7.1. Causal Depth Requires Molecular Epistasis and Separation-of-Function Rescue

The causal hierarchical organization of the system described in this section can be characterized by seven levels of abstraction:

(1) A chromatin injury → (2) A particular retroelement locus → (3) The first molecular intermediate → (4) A restriction/sensor pathway → (5) A persistent cellular state → (6) Intercellular effector molecules → (7) Final circuit phenotype.

Therefore, the nature of an intervention at any given level of the hierarchy will depend on where in the hierarchy that intervention is being applied [214]. Thus, rescuing the activity of SETDB1 will determine whether there is a chromatin dependency; removing the expression of one particular ERV or LINE-1 locus will determine whether there is a locus dependency; blocking reverse transcription will determine whether RNA-dependent effects of retroelements are separable from effects due to DNA-containing intermediates; using an RNase H-dependent rescue will determine whether the relevant intermediate is an RNA–DNA hybrid; disrupting a sensor component will determine which pathway recognizes the presence of a candidate retroelement-derived molecular species; disrupting PML will determine whether maintenance of a persistent cellular state is dependent on PML; finally, depleting complement, depleting microglia, or disrupting neuronal complement-associated trafficking will determine whether the ultimate phenotypic defect is dependent on these downstream effector mechanisms [215].

Separation-of-function alleles are uniquely valuable because many proteins involved in this pathway perform multiple functions. Evaluating whether SETDB1 function can be restored by comparing mutants that preserve protein levels but disrupt specified molecular interfaces or catalytic activities will therefore be particularly informative. The triple-Tudor domain of SETDB1, including the F332-associated recognition site, presents an example of an interface that lends itself to this type of analysis [216,217]. Mutants of this domain will enable determination of whether restoration of H3K9me3 at LINE loci depends on this recognition mechanism even though SETDB1 protein levels are preserved. Likewise, STING variants affecting E282/D283 will enable separation of ORF1p trafficking from many other canonical signaling events; mutants of ORF2p lacking endonuclease activity or reverse-transcriptase activity will enable separation of genomic nicking from cDNA synthesis; mutants of PML that fail to oligomerize, undergo SUMOylation, or bind client proteins will enable separation of nuclear-body assembly from preservation of PML protein levels; and rescue with SNX27 will enable separation of receptor-trafficking defects from extracellular complement activity. Confidence in attributing mechanisms to phenotypic defects increases when it can be shown that a given phenotypic abnormality arose as a result of preventing a well-defined biochemical process from occurring rather than as a result of complete loss of a pleiotropic protein [218].

However, disrupting a biochemical process does not demonstrate whether the disruption prevented an event from occurring or reversed an event that had already occurred. Therefore, correction of the original injury should occur at different points in time: before retroelements have escaped repression; after ligand formation has taken place; and after a persistent cellular state has been established. If eliminating upstream disruptions leads to complete elimination of downstream phenotypic abnormalities, continued dependency is implied. However, if the initiating molecular intermediate has disappeared but phenotypes related to checkpoints, PML, or the persistent microglial state remain, this would provide evidence consistent with hysteresis, particularly if path dependence is also demonstrated [219].

Therefore, the most robust approach will involve bidirectional epistasis. Downstream disruptions should remove downstream phenotypes while preserving upstream abnormalities; conversely, upstream manipulations should sequentially remove downstream states. If disrupting reverse transcription improves circuit function without changing the RNA–DNA hybrid levels presumed to mediate the signal, then ambiguity regarding attribution of causality to that RNA–DNA hybrid is created. Conversely, if selectively removing hybrids eliminates ERV-induced complement activation while ERV-RNA levels remain unchanged, then causality can be attributed more specifically to the physical molecular state generated downstream of transcription rather than to transcription per se [220].

### 7.2. Locus- and Structure-Resolved Measurements Should Replace Generic “TE Expression”

To address the limitations listed above, we recommend a hierarchical analytical paradigm that addresses each issue identified; this can be viewed as a “graded” approach. Therefore, we recommend replacing generic TE-expression data with locus-, structure-, and time-resolved data. For instance, reads assigned to a particular repeat family could potentially represent multiple biologically distinct processes, including autonomous transcription of the repeat itself; host-gene readthrough; sequences contained within an intron; antisense transcription of the repeat; and transcription primarily derived from a few highly active loci. These ambiguities are exacerbated when analyzing young repeats containing high degrees of sequence identity and therefore substantial multimappability, particularly among those copies retaining the greatest functional capacity [221].

Because long-read transcriptomics allows assignment of reads to individual loci based on unique flanking sequences and/or transcript architectures, combined with the increased sensitivity provided by integration with deep short-read sequencing, locus specificity should always include mapping of transcription start sites. Techniques including CAGE, RAMPAGE, and 5′ RACE will allow determination of whether transcription initiates within the retroelement itself versus entry through adjacent sequence. Similarly, use of strand-specific library preparation will enable distinction among sense, antisense, and readthrough transcription. As such, the basic unit of analysis becomes an autonomously initiated transcript derived from a specified genomic copy [222].

Another major limitation imposed by reference-genome assemblies involves presence/absence polymorphism among actively transcribing human LINE-1, Alu, and SVA insertions. When locus specificity is essential to understanding mechanism, particularly in the human brain where non-reference insertions may remain undetected or incorrectly assigned, matched long-read genomic sequencing is preferred [223].

Because full-length human LINE-1 copies with potential retrotransposition competence constitute only a small proportion of total genomic LINE-1 sequence, family-level measurements may substantially overestimate the number of loci capable of undergoing subsequent molecular transitions [224].

Similarly, single-cell analysis will need to distinguish among these ambiguous possibilities instead of ignoring them. Machine-learning-based computational methods utilizing locus-specific flanking sequences or local coverage patterns can recover portions of multimappable information across scRNA-seq, scATAC-seq, and paired multiomic datasets. Regardless, accessibility, transcriptional capability, and molecular activity remain separate variables. Thus, an accessible repeat does not necessarily mean that it is being transcribed; nor does transcription mean that it produces a structurally competent product or encodes proteins capable of downstream enzymatic activity [225].

Thus, chromatin profiling should also be locus resolved. Use of CUT&RUN or CUT&Tag for H3K9me3, H3.3, SETDB1, TRIM28, H3K4me3, and H3K27ac, together with chromatin-accessibility mapping and transcription-start-site mapping, can define at least four experimentally relevant states: repressed; accessible-but-repressed; autonomously transcribed; and regulatory-element-like [226].

Following transcription, the analytical standard must be structural. Instead of inferring dsRNA through bidirectional expression alone, evidence directly demonstrating dsRNA must be presented. Ideally, dsRNA should be demonstrated using duplex enrichment, strand resolution during sequencing, and nuclease controls enabling differentiation among dsRNA, ssRNA, and DNA. Additionally, adenosine-to-inosine (A-to-I) RNA-editing maps can provide insight into ADAR processing. Purifying receptor-associated RNA represents stronger evidence for functional interaction than simply detecting elevated sensor expression or colocalization [227].

Similar rigor must exist for hybrid structures. Demonstrating the existence of a defined RNA–DNA duplex or canonical R-loop through S9.6 reactivity alone is insufficient. Hybrid assignment must demonstrate RNase H dependence and should be supported by an independent or orthogonal hybrid-detection method. To characterize a structure as a canonical R-loop, evidence demonstrating hybridization of RNA to genomic DNA with concomitant displacement of the complementary DNA strand must exist. Genomic-coordinate information, strand orientation, enzymatic dependence, and structural controls must therefore accompany mechanistic statements relating to retroelement-associated hybridization [228].

There is a strict hierarchy in LINE-1 analyses:

RNA ≠ ORF1p ≠ ORF2p ≠ reverse transcription ≠ insertion.

Targeted proteomics can measure endogenously expressed ORF1p and the much less abundant ORF2p, although detecting endogenous ORF2p remains technically difficult. Sequence architecture, subcellular fractionation, and nuclease-sensitive controls can provide evidence distinguishing reverse-transcription products from pre-existing genomic LINE-1 DNA. Because canonical target-primed reverse transcription begins at chromosomal target sites, reverse-transcription products cannot automatically be assumed to constitute cytoplasmic DNA. Completion of retrotransposition requires demonstration of a new genomic insertion with mapped junctions, a poly(A) tract, and target-site duplication. Long-read DNA sequencing provides a strong method for demonstrating completion of retrotransposition [229].

Consequently, a chain of evidence must be reconstructed around the experimental object:

locus → autonomous transcript → molecular structure → protein/RNP state → enzymatic product → sensor engagement → cellular response.

No single assay can demonstrate this chain of evidence. The strength of an analysis is determined by concordance among multiple assays addressing different physical aspects of the same process [230]. Table 4 summarizes the causal tests needed to progress from locus-resolved chromatin escape to structurally defined intermediates, direct sensor engagement, persistent-state formation, and ultimately circuit dysfunction.

### 7.3. The Decisive Experiments Should Isolate Molecular Conversions

For us to understand whether a causal link exists between retroelements and their effects on neural function, we must determine the molecular conversions, for example RNA to DNA, associated with ERVs and LINE-1 elements [239].

Future research will therefore need to focus on the effects of specifically disabling individual transitions of retroelements, such as turning off a specific LINE-1 or ERV locus. For instance, researchers could delete or selectively inactivate a particular LINE-1 element using CRISPR technology to determine whether the activity of merely a few LINE-1 copies is sufficient to generate a substantial amount of downstream pathology [240].

Our second goal is to determine molecular-species epistasis. We define molecular-species epistasis as demonstration that an effect of LINE-1 or ERV transcription is due to a defined molecular product being recognized by immune-surveillance pathways, as opposed to simply being due to transcription itself. As proposed, researchers should first confirm that a candidate locus remains transcriptionally active while preventing formation of at least one possible downstream product generated from that locus. There are several ways in which researchers can carry out this task [241].

There are three different approaches that researchers could undertake to accomplish this. One approach is to introduce mutations designed to prevent formation or persistence of candidate nucleic-acid structures while preserving transcription. Another approach is to disrupt RNA–DNA hybrids produced by retroelements using targeted RNase H activity. A third alternative is to express LINE-1 with either endonuclease-deficient ORF2p or reverse-transcriptase-deficient ORF2p. If eliminating the potential downstream products eliminates the observed phenotypic response while leaving transcript levels unchanged, researchers will have demonstrated that the observed effect depends upon the molecular composition or state of the downstream product resulting from transcription rather than simply on the act of transcription itself [242].

Following identification of sensor assignment as a ligand-by-sensor matrix, researchers should next evaluate how the same molecular species behaves under varying conditions while independently disrupting individual members of the innate immune system, including MDA5, RIG-I, cGAS, and ZBP1, along with their relevant adaptor pathways, including MAVS and STING. Our model predicts that these surveillance pathways recognize and respond differently to distinct physical forms of nucleic acids resulting from retroelement activity. Failure to demonstrate the expected pattern of responses would therefore provide evidence against direct ligand–sensor assignment and in favor of indirect inflammatory cross-talk [243].

Demonstrating hysteresis requires a temporal reversal experiment. Researchers should allow a persistent phenotype to emerge before correcting the original genetic lesion. They should then follow the clearance of retroelement-derived products and the subsequent resolution of checkpoint activation, PML organization, and microglial state over time. Persistence of downstream behavior after validated normalization of the upstream lesion would provide evidence of molecular memory, whereas rapid return toward the previous state following normalization would indicate continued dependence upon upstream factors and argue against hysteresis. Demonstration of hysteresis also requires evidence of path dependence in addition to persistence [244].

Because active retroelement repertoires and lineage-specific ERV architectures differ greatly among mammals, studies utilizing human cells and tissues will be necessary to determine which mechanistic principles established in experimental models are conserved and which loci are species restricted. Ideally, researchers should utilize iPSC-derived human neurons and microglia together with long-read genomic and transcriptomic data, locus-resolved single-cell RNA and ATAC analyses, structure-specific nucleic-acid assays, and synaptic or electrophysiological measurements. After these studies are conducted in experimentally tractable systems, researchers can evaluate spatial organization, molecular coexistence, and disease relevance in human tissue samples; however, observation in human tissue cannot substitute for causal experimentation [245].

Ultimately, support for a retroelement-based mechanism requires defining which genomic copy of which retroelement is involved; demonstrating autonomous transcription; recovering and characterizing the relevant molecular species; demonstrating that the enzymatic process producing that species is necessary; demonstrating that downstream recognition and effector mechanisms are organized through hierarchical epistasis; and, ultimately, demonstrating that restoration of normal neuronal function occurs through selective inhibition of the relevant molecular conversion rather than through nonspecific reduction in inflammation.

## 8. Conclusions—The Genome–Immunity Boundary

Increasingly, the “repetitive genome” is being understood as a physically regulated compartment with important implications for both neuroimmune function in the brain and immune homeostasis outside the brain. In addition to a number of other molecular checkpoints, many of which act sequentially, there are several checkpoints that control the physical compartmentalization of this genome. Specifically, these checkpoints include repression of chromatin, RNA processing, structural regulation of processed RNAs, regulation of RNP formation, restriction of reverse transcription, degradation of aberrant intermediate nucleic-acid structures, and termination of the cellular state established as a result of the generation of those structures. As a consequence, pathology may occur when one or more of these checkpoints fail to prevent endogenous sequences from acquiring physical molecular characteristics that are recognizable as foreign by the host’s surveillance systems.

It is therefore essential that we redirect our focus of study away from repeat families toward specific molecular states that are established independently by each locus. Unexpressed ERVs, autonomously transcribed ERVs, ERV-derived double-stranded RNA molecules, RNA–DNA hybrids, LINE-1 RNA molecules, ORF1p-containing RNPs, ORF2p activity, recently synthesized cDNA, and fully integrated new genomic elements are examples of discrete biochemical states with unique lifespans, subcellular locations, and capacities to contribute to pathological processes. Consequently, the most relevant biological parameters are the frequencies at which individual loci transition through these states and the probability that each transition will circumvent host restriction.

Furthermore, this approach provides insight into how widespread disruptions in genome regulation can produce highly localized neuronal and synaptic pathology. Pathological signals become progressively filtered depending on their location in space and time; specifically, by locus architecture, nucleic-acid topology, nucleic-acid-associated proteins, subcellular location, proximity to sensors, cell type/state, complement competence, and local circuit organization. It is thus possible that circuit specificity develops as a result of sequential molecular filtering of a lesion site rather than spatially restricted initiation of the original genomic defect.

The initial mechanisms that lead to an abnormal state in cells do not have to be the same mechanisms responsible for maintaining that abnormal state. Candidate mechanisms that can be involved in sustaining long-term disruption include alterations to the DNA-damage response; changes to PML (promyelocytic leukemia) nuclear organization; changes to gene expression through transcriptional remodeling; changes in lysosomal function; and changes in metabolism. The relative importance and necessity of these mechanisms are likely to vary depending on the specific context. Rather than identifying candidates for clinical trials, the distinctions identified provide a basis for generating hypotheses that could be tested in preclinical models prior to initiating clinical trials. Interventions aimed at restoring repression of genes by chromatin, inhibiting reverse transcription of viral RNA into DNA, affecting how nucleic acids are processed, activating or suppressing innate immune sensor proteins, disrupting persistent-state mechanisms, or disrupting microglial and neuronal effectors that rely on complement activation occur at different levels of the proposed cascade. Thus, it would seem logical that interventions targeting each level should vary based upon the disease stage and the specific cell types involved. None of the potential targets identified above has been shown by the data presented here to represent a disease-modifying therapy for either human neuroinflammatory or neurodegenerative diseases. Ultimately, comparative studies examining timing, specificity to particular cell types, target engagement, reversibility, efficacy, and safety will need to be completed before therapeutic options can be rationally chosen.

More rigorous experimentation will be required to test this conceptual framework. Current terminology utilized to describe the various molecular events, including “activation,” “viral mimicry,” and “pruning,” is inadequate as a description of causal events unless sufficient detail concerning the responsible locus, the autonomous transcript produced from that locus, the physical forms of nucleic acids produced from that transcript, the enzymes involved in producing those physical forms, and the sensors/restriction mechanisms recognizing those physical forms is supplied. Available technologies such as single-cell and long-read sequencing, as well as structure-specific nucleic-acid mapping methods, can be employed to address some of these issues. Separation-of-function genetics and temporally resolved studies assessing genetic interactions (epistasis) will enable investigators to disentangle these variables. Understanding which elements reach which molecular states, at which checkpoints transitions between these states are prevented, and which transitions remain reversible will likely provide greater insight into disease etiology than simply documenting increased levels of retroelement expression in disease.

Finally, retroelement neuroimmunology can be considered as a problem of molecular identity. The central nervous system must constantly retain repetitive nucleotide sequences as genomic self despite the fact that subsets of those sequences can generate molecular structures similar to those generated during viral replication. While sequence origin is inadequate to define whether an endogenous nucleic acid will be recognized by innate immunity or remain tolerated, a combination of chromatin structure, nucleic-acid topology, associated proteins, subcellular localization, sensor accessibility, and prior cellular state can affect whether endogenously generated nucleic acids remain undetectable by innate surveillance systems or become detectable.

Three areas of investigation immediately follow logically from this conceptualization: locus-defined identification of the retroelements responsible for generating pathogenic molecular species; evidence supporting physical ligand–sensor interactions responsible for innate recognition; and temporally controlled reversals to demonstrate whether continued reliance on the initiating lesion or progression through subsequent steps in pathogenesis leads to persistent cellular dysfunction. Together, these studies should provide sufficient detail to establish causal links between failures in chromatin regulation leading to molecular-state transitions; innate surveillance; microglial dysfunction; complement-mediated intercellular signaling; and selectivity in injury to local circuits.

Another potential area of research in retroelement neuroimmunology may therefore not lie solely in providing additional descriptions of families of derepressed retroelements, but rather in identifying the specific molecular event or events by which a genomic self-sequence is converted into immunogenic material—and determining under what conditions that event generates persistent dysfunction in neurons and local circuits. Defining boundaries around these areas could provide a unified framework linking chromatin biology, nucleic-acid chemistry, innate immune surveillance, and selective neural-circuit pathology.

In summary, the above conceptualization represents a basic principle applicable across individual retroelements and neurological disorders: pathological consequences are not solely a result of alterations in genomic informational content, but may also arise when molecular systems that normally recognize, process, and compartmentalize that informational content as self-fail.

## Figures and Tables

**Table 1:** Traces the progression from ADD-domain multiligand recognition and CHD4 molecular mimicry to DAXX-directed H3.3 deposition, SETDB1/SUV39-dependent H3K9me3 renewal, and repeat-specific silencing, highlighting how defective renewal may preserve nucleosome occupancy while permitting retroelement transcription and viral mimicry. Evidence levels distinguish direct mechanistic evidence (Level I), functional or perturbational evidence (Level II), and context-dependent or inferential evidence (Level III).

Regulatory Layer	Core Machinery	Molecular Mechanism	Chromatin Consequence	Mechanistic Interpretation	Evidence Level	References
Combinatorial recognition	ATRX ADD; H3K4me0; H3K9me3	Simultaneous recognition of unmethylated H3K4 and trimethylated H3K9	Preferential ATRX docking at repressive nucleosomes	Repressive identity is encoded combinatorially rather than by a single histone mark	Level I	[50]
Multiligand recognition	ATRX ADD; macroH2A	macroH2A R40 engages a surface overlapping the H3K9me3-recognition region; ATRX Y203, H189, and E252 contribute to binding	Variant-sensitive ATRX residence	Histone tails and histone variants can converge on overlapping reader surfaces	Level I	[51]
Molecular mimicry	CHD4/NuRD; ATRX	CHD4 K261 occupies an H3K4-like position; R271 approximates the H3K9-recognition environment	Remodeler–remodeler coupling	Non-histone ligands may contribute to chromatin-state recognition	Level III	[52,53]
ATP-dependent remodeling	ATRX SNF2 ATPase	Chromatin recognition is coupled to ATP-dependent remodeling at repeat-rich loci	Repressive nucleosome organization	ATRX integrates reader and remodeler functions	Level I	[54]
H3.3 deposition	DAXX; H3.3–H4	DAXX selectively packages and deposits H3.3–H4 dimers	Replication-independent histone renewal	Histone turnover can occur without loss of chromatin identity	Level I	[55]
Chaperone specificity	DAXX versus HIRA	DAXX loss decreases H3K9me3 despite comparatively preserved H3.3; HIRA loss is non-equivalent	Chaperone-dependent chromatin state	Chromatin state depends partly on the context of histone deposition	Level II	[56]
H3K9me3 renewal	DAXX; SETDB1; SUV39H1/2	Histone delivery is coupled to repressive H3K9 methyltransferase activity	Restoration of H3K9me3 after histone exchange	Heterochromatin identity is supported by continuous biochemical renewal	Level II	[57]
G4-associated protection	ATRX; DAXX; H3.3; SETDB1/ESET	ATRX associates with G4-prone DNA; ATRX/DAXX-dependent H3.3 deposition supports H3K9me3-rich closed chromatin and limits G4-associated replication stress	Reduced accessibility and structural stress at G4-rich loci	ATRX–DAXX-associated repression may extend protective chromatin organization to structurally challenging DNA	Level II	[46]
Repeat-family selectivity	ATRX–DAXX; KRAB-ZFP/TRIM28; DNA methylation	Repeat families differ in dependence according to age, promoter architecture, CpG content, and local regulatory context	Unequal repression among repeats	Silencing is locus- and context-conditioned rather than uniform	Level II	[58]
State decoupling	DAXX; H3.3; H3K9me3	H3.3 can remain while H3K9me3 declines	Nucleosome occupancy is preserved while biochemical identity is altered	Loss of repressive chromatin identity can precede nucleosome loss	Level II	[59]
Immune permissiveness	Derepressed ERVs/LTRs; structured nucleic acids	Failure of repressive renewal can increase repeat transcription and formation of potentially immunogenic molecular species	Increased potential for viral mimicry	Epigenetic failure may provide an upstream source of candidate neuroimmune ligands	Level III	[60]

**Table 2:** Structural determinants relevant to MDA5, RIG-I, cGAS, and ZBP1 surveillance of candidate retroelement-derived nucleic acids, together with requirements for sensor attribution and principal interpretive limitations. Evidence levels distinguish direct sensor-recognition mechanisms (Level I) from functional or context-dependent retroelement-specific attribution (Levels II–III).

Sensor	Preferred Molecular Determinant	Principal Recognition Geometry	Downstream Signaling Axis	Evidence Required for Retroelement Attribution	Interpretive Limitation	Evidence Level
MDA5	Extended accessible dsRNA	Internal duplex continuity; cooperative filament formation	MDA5–MAVS	Sensor-associated RNA, structural validation, and MDA5 dependence	Repeat RNA abundance alone does not demonstrate MDA5 engagement	Level I/II–III *
RIG-I	Accessible duplex termini with compatible 5′ chemistry	Terminal geometry and end accessibility	RIG-I–MAVS	Terminal characterization, RIG-I dependence, and preferably direct association	Compatible termini may remain inaccessible because of protein occupancy	Level I/II–III *
cGAS	Sufficiently exposed DNA	DNA surface supporting productive cGAS assembly	cGAS–cGAMP–STING	Appropriate compartmentalization, cGAS-associated DNA, and/or genetic epistasis	Reverse transcription or cDNA accumulation alone does not establish cGAS sensing	Level I/II–III *
ZBP1	Z-RNA or Z-DNA	Left-handed Z-conformation recognized by Zα domains	ZBP1–RHIM–RIPK3/other context-dependent outputs	Demonstration of Z-form nucleic acid, Zα dependence, and ZBP1 genetic requirement	ZBP1 activation does not invariably imply RIPK3–MLKL necroptosis	Level I/II–III *

**Table 3:** Sequential checkpoints linking LINE-1 cargo handling, reverse transcription, DNA visibility, cGAS–STING signaling, and interferon-dependent restriction. Evidence levels distinguish direct mechanistic evidence (Level I), functional or perturbational evidence (Level II), context-dependent or inferential evidence (Level III), and proposed conceptual synthesis (Level IV).

Checkpoint	Molecular Determinant	Mechanistic Event	Diagnostic Readout	Interpretive Value	Evidence Level	References
ORF1p capture	STING dimer; LINE-1 ORF1p	STING–ORF1p association before extensive reverse transcription	ORF1p–STING co-IP; ORF1p abundance	Supports a proposed pre-sensor restriction step	Level II	[19]
Sorting competence	STING E282/D283; dimer interface	Cargo-selective trafficking; E296/D297 dispensable	Mutant-rescue trafficking assay	Supports localization of restriction to a discrete acidic determinant	Level II	[148]
Endolysosomal disposal	ER, ERGIC, Golgi, Rab7, LAMP1	ORF1p-containing material is routed toward late endosomes/lysosomes	Rab7/LAMP1 colocalization; ORF1p half-life	Links STING-associated cargo routing to retroelement clearance	Level II	[149]
Noncanonical degradation	ATG5, ULK1, ATG9A, p62, Beclin1; TSG101, ALIX	Restriction persists despite disruption of tested macroautophagy/ESCRT components	Preserved ORF1p depletion after depletion/knockout	Indicates that the precise trafficking mechanism remains unresolved	Level II	[150]
Reverse-transcription control	ORF1p/ORF2p machinery	Reduced RNP persistence limits cDNA generation	LINE-1 cDNA; retrotransposition reporter	Supports the possibility that source restriction can precede ligand sensing	Level II	[151]
DNA visibility gate	Nucleases, chromatinization, DNA-binding proteins	Only appropriately exposed and compartmentalized DNA supports productive cGAS assembly	Accessible DNA; cGAS-associated DNA	Separates DNA abundance from demonstrated sensor competence	Level III	[152]
cGAS catalytic activation	cGAS, ATP, GTP	DNA-dependent synthesis of 2′3′-cGAMP	cGAMP abundance; cGAS assembly	Converts appropriately exposed DNA into second-messenger information	Level I	[153]
STING signaling output	cGAMP–STING, TBK1, IRF3, NF-κB	cGAMP-bound STING activates canonical downstream signaling	p-STING; p-TBK1; p-IRF3; IFNB1	Separates canonical signaling from ORF1p cargo handling	Level I	[140]
LINE-1 transcriptional feedback	cGAS, CTCF, RUNX3	cGAS activity modifies human LINE-1 transcriptional competence	LINE-1 RNA; promoter occupancy	Innate signaling can alter future ligand production; species dependence remains important	Level II	[154]
IFN receptor amplification	IFNAR1/2, JAK1, TYK2	IFN-I converts sensing into a broader restriction program	p-JAK1/TYK2; p-STAT1/2	Establishes feedback amplification	Level I	[155]
ISGF3 restriction module	STAT1, STAT2, IRF9	Induction of editors, nucleases, helicases, and repeat suppressors	ISG score; ADAR1, DAXX, TREX1 expression	Raises the threshold for subsequent ligand escape	Level I	[156]
Containment recovery	ADAR1, DAXX, TREX1, helicases, lysosomal flux	Editing, degradation, repression, and trafficking reduce ligand persistence	Repeat RNA/DNA burden; editing rate	Consistent with successful negative feedback	Level III	[157]
Feedback uncoupling	Persistent DNA damage, p53/p21 and cell-state programs, chromatin failure	ISG output falls after IFN blockade while lesion-associated programs persist	Reduced ISGs; persistent DNA-damage/p53/p21/state markers	Distinguishes the inflammatory response from the underlying lesion	Level II	[158]
Homeostatic STING branch	STING-associated genome/tissue maintenance	Surveillance contributes to genomic stability and barrier integrity in defined CNS models	DNA-damage burden; BBB metrics; microglial state	May help explain why STING loss worsens CNS homeostasis in some contexts	Level II	[159]
Chronic inflammatory branch	Persistent cGAS–STING–TBK1 signaling	Sustained activation maintains inflammatory transcription	Sustained p-TBK1/p-IRF3; chronic ISGs	The same node may become pathogenic when persistent signaling predominates	Level II	[140]
Flux-over-capacity transition	Ligand production versus editing, degradation, shielding, and trafficking	Continuing ligand generation may exceed effective restriction capacity	Longitudinal ligand burden/clearance; sustained pathway activation	Proposed conceptual transition from containment to persistent activation; not a universal stoichiometric threshold	Level IV	[160]

**Table 4:** Proposed multilevel experimental framework for increasing causal resolution in retroelement-associated neuroimmune pathology, linking locus-resolved chromatin escape and structurally defined nucleic-acid intermediates to enzymatic competence, candidate sensor engagement, persistent-state formation, and circuit dysfunction, with emphasis on orthogonal validation, separation-of-function rescue, and bidirectional epistasis. The framework is classified as Level IV evidence (proposed conceptual/experimental synthesis) and should not be interpreted as an established methodological standard.

Proposed Mechanistic Question	Most Informative Causal Test	Separation-of-Function Strategy	Required Orthogonal Evidence	Key Interpretive Safeguard	References
Does a chromatin lesion initiate escape?	Restore SETDB1 before TE activation	Catalytic-dead vs. reader/Tudor-defective rescue	Locus-resolved H3K9me3, H3.3, SETDB1/TRIM28 occupancy	Global heterochromatin loss does not prove locus causality	[65]
Is a specific TE locus causal?	Silence or excise one active copy	CRISPRi, allele-specific ASO, locus-specific deletion	Long-read locus assignment, TSS, strand, flanking sequence	Family-level RNA abundance can mask a few dominant loci	[231]
Does autonomous transcription occur?	Disrupt internal promoter/TSS	Promoter-specific editing preserving host gene	CAGE, RAMPAGE, 5′ RACE, strand-specific RNA-seq	Distinguish autonomous initiation from readthrough/intronic RNA	[232]
Is dsRNA the active intermediate?	Selectively remove or destabilize duplex	Duplex-forming-sequence mutant or dsRNase-sensitive rescue	Duplex enrichment, strand pairing, A-to-I editing, nuclease controls	Bidirectional transcription alone is insufficient	[233]
Is an RNA–DNA hybrid causal?	RNase H-dependent rescue	Active vs. catalytic-dead RNase H	Orthogonal hybrid detection, genomic coordinate, strand assignment	S9.6 alone does not establish an R-loop	[234]
Are LINE-1 proteins required?	Deplete ORF1p and ORF2p independently	ORF2p endonuclease-dead vs. RT-dead alleles	Targeted proteomics, RNP localization, RNP assembly	LINE-1 RNA does not imply enzymatic competence	[102]
Does reverse transcription contribute to the phenotype?	RT inhibition with upstream RNA preserved	Catalytic ORF2p mutants or orthogonal RT inhibitors	Reverse-transcription-product architecture, fractionation, nuclease sensitivity	Separate cDNA from pre-existing genomic LINE-1 DNA	[151]
Is retrotransposition completed?	Map de novo insertion	Junction-specific validation	5′/3′ junctions, poly(A), target-site duplication	cDNA accumulation is not equivalent to insertion	[235]
Is sensor engagement direct?	Remove receptor while retaining ligand	Binding-deficient vs. signaling-deficient sensor allele	Receptor-associated nucleic acid; unchanged upstream ligand burden	Expression or colocalization does not prove engagement	[236]
Does the sensor branch determine the downstream output?	Genetic epistasis across sensor and adaptor nodes	Domain-specific mutants in MDA5, RIG-I, cGAS, ZBP1, MAVS, STING	Ligand persistence plus branch-selective signaling loss	Cytokine induction alone cannot assign pathway identity	[237]
Does the persistent state demonstrate path dependence?	Normalize the initiating lesion after state establishment	Inducible rescue; PML oligomerization/SUMO/client-binding mutants	Upstream intermediate resolves while checkpoint/state markers persist	Delayed normalization alone does not demonstrate hysteresis	[238]
Does the terminal effector contribute causally to circuit injury?	Block complement, microglia, or trafficking while the lesion persists	Effector-specific rescue or depletion	Upstream molecular lesion retained; circuit phenotype improved	Distinguish initiation from execution	[65]
Does bidirectional epistasis support causal ordering?	Upstream rescue eliminates downstream effects; downstream rescue spares upstream abnormalities	Reversible, temporally staged multi-node perturbation	Simultaneous molecular, cellular, and circuit readouts	Static association cannot establish causal order	[231]
Does the mechanism generalize across experimental contexts?	Replicate causal ordering across models/species	Independent alleles and orthogonal perturbations	Convergent genomic, structural, proteomic, and functional endpoints	Distinguish conserved mechanisms from species-specific loci	[232]
Has the mechanistic claim reached high causal resolution?	Concordant perturbation, rescue, and temporal ordering	At least one separation-of-function allele	Locus + structure + protein/RNP + product + sensor + phenotype	No single assay establishes the complete causal chain	[233]

## Data Availability

No new data were created or analyzed in this study. Data sharing is not applicable to this review article.
